# Metformin modulates the phenotypes of tumor-associated macrophages in glioblastoma in a context-dependent manner by promoting the homeostasis of macrophages

**DOI:** 10.3389/fonc.2026.1834973

**Published:** 2026-08-14

**Authors:** Ashish Das, Sadaf Mahfooz, Fei Wang, Bingjie Guan, Benhua Xu, Chi Zhang, Zi Wang, Ghanbar Mahmoodi Chalbatani, Yuanyuan Jia, Chi Lin, Chi (Kevin) Zhang

**Affiliations:** 1 Department of Pathology, Microbiology, and Immunology, University of Nebraska Medical Center, Omaha, NE, United States; 2 Department of Radiation Oncology, Mayo Clinic Arizona, Phoenix, AZ, United States; 3 Department of Radiation Oncology, Fred & Pamela Buffett Cancer Center, University of Nebraska Medical Center, Omaha, NE, United States; 4 Department of Radiation Oncology, United Hospital of Fujian, Fuzhou, Fujian, China; 5 School of Biological Sciences, University of Nebraska, Lincoln, Lincoln, NE, United States

**Keywords:** glioblastoma, homeostasis, immune checkpoint, immunotherapy, metformin, myeloid-derived suppressor cells, radiation therapy, tumor microenvironment

## Abstract

**Background:**

Glioblastoma (GBM) remains the most aggressive primary adult brain cancer attributed to its immunosuppressive nature. Radiation therapy (RT) and concurrent temozolomide chemotherapy are the standard treatments for GBM. Emerging evidence indicates that metformin has potential as an anti-tumor agent that can reshape the immune landscape across various malignancies. We thus postulate that metformin may enhance the anti-tumor effect of RT by modulating the immunosuppressive milieu in GBM.

**Methods:**

We first explore multiple *in vitro* conditions, with or without pre-induction, and mimicked a tumor microenvironment to demonstrate a highly context-dependent effect of metformin on the polarization of bone marrow-derived macrophages (BMDMs). We then investigated the antitumor activity and immune-modulatory effects of metformin in combination with RT in GBM-bearing mice.

**Results:**

The *in vitro* experiment showed that metformin inhibited the immunosuppressive effects of IL-4/IL-13 but also the immunostimulatory effects of Lipopolysaccharide (LPS) on BMDMs, demonstrating bidirectional immunomodulatory properties that depend on the baseline inflammatory stimulus. In tumor cell co-culture environment, metformin exhibited context-dependent immunoregulatory effects, predominantly promoting M1-associated activation. Notably, concurrent metformin treatment counteracted M2 polarization typically induced by the tumor microenvironment. In a high m-MCSF inducted M2-dominated condition, metformin preferentially shifted the highly polarized M2 phenotype toward an M1-like state in a time and dose-dependent manner but with limited promotion of M1 subtype maturation. RNAseq results on BMDM treated with metformin showed increased homeostasis. Metformin showed dose-dependent immunomodulatory effects when combined with RT *in vitro*. In syngeneic GBM mouse models, concurrent metformin + RT significantly prolonged survival and reduced tumor burden by reprogramming tumor-associated macrophages (TAMs), elevating intratumoral CD8+ T-cell infiltration and the CD8+/Treg ratio, increasing circulating CD8+ T cells, reversing RT-induced expansion of monocytic myeloid-derived suppressor cells (mMDSCs), and expanding CD4+ and CD8+ effector memory populations in peripheral blood.

**Conclusion:**

Metformin demonstrates inhibitory effects on M2 phenotype of macrophages. However, it is not a simple M2 inhibitor; instead, it functions primarily as a neutralizer of highly polarized macrophage states by promoting transcriptomic homeostasis. This context-dependent activity enables metformin to relieve the profoundly immunosuppressive TME of GBM to potentiate the anti-tumor effects of RT and by enhancing systemic immune memory.

## 1 Introduction

Glioblastoma (GBM) is the most common and lethal form of primary brain malignancy in adults, with an associated median survival of less than 2 years, despite extensive research and progress in therapies for the treatment of GBM over the last several decades (1). A central barrier to durable treatment responses in GBM is the profoundly immunosuppressive tumor microenvironment (TME), characterized by the accumulation of immunosuppressive cell populations in proximity to the tumor and the heightened expression of immunosuppressive molecules and their cognate receptors (2, 3).Glioblastoma-associated microglia and macrophages (GAMs), the TAMs in GBM, constitute the predominant immune cell population within GBM, comprising up to 50% of the tumor cellular mass (4). Accumulating evidence indicates that GAM polarization plays an essential role in the growth and progression of GBM (5). GAMs exist across a phenotypic spectrum, which can be broadly classified into two functional subtypes: proinflammatory/antitumor M1 (M1*Φ*) and the alternatively activated M2 phenotypes (M2*Φ*) that promote tumor growth, which establish an immunosuppressive TME in GBM (6). Emerging data indicates that the GAMs within GBM comprises functionally distinct populations that frequently display characteristics of both M1 and M2 phenotypes yet collectively demonstrate greater similarity to alternatively activated macrophages (7). Inactivation of tumor suppressor genes (e.g., PTEN, NF1, and TP53) in GBM cells recruits macrophages and microglia into the GBM TME where they are subsequently polarized into an M2-like phenotype in mouse and human GBM models (8). Once polarized, TAMs promote GBM progression through multiple mechanisms, including the production of immunosuppressive molecules, suppression of T cell activation and proliferation, and promotion of brain tumor angiogenesis, thereby facilitating tumor cell colonization and outgrowth (9). Contemporary evidence, however, reveals that TAMs exhibit substantial phenotypic plasticity, enabling dynamic adaptation to diverse microenvironmental signals in tumors including GBM from preclinical studies (10, 11).

In addition to its interaction with GAM, GBM induces systemic immunotolerance through deletion and impaired T-cell cytotoxicity against multiple tumor antigens, with AIDS-level CD4 counts in the brain and correlated T-cell deficiency in contracted lymphoid organs (12). Accumulating evidence demonstrates that depleted T cells become extensively sequestered within the bone marrow compartment consequent to dysregulated sphingosine-1-phosphate receptor 1 (S1P1) signaling on the T cell surface (13, 14). On top of CD4+ T cells depletion, the tumor-infiltrating CD8+ cytotoxic T lymphocytes (CTL) are also found to have impaired effector functions and an exhausted phenotype, resulting in an ineffective role as cytotoxic lymphocytes in GBM (15, 16). Regulatory T cells, a CD4^+^ subset whose abundance correlates directly with tumor grade, constitute another pivotal mechanism sustaining the immunologically hostile glioblastoma microenvironment (17).

Radiation therapy (RT) is indispensable in the treatment of all GBM patients. However, evidence shows that RT itself could induce a rapid inflammatory response leading to the recruitment of TAMs, increasing the proportion of M2 phenotype macrophage and activating multiple pathways, which potentially contribute to tumor progression and recurrence (5, 18). To enhance tumor control in glioblastoma, it is essential to address the immunosuppressive tumor microenvironment and reverse the systemic immune dysfunction induced by radiation therapy.

Metformin (MET), 1, 1-dimethylbiguanide hydrochloride, is a biguanide drug prescribed as a first-line medication for type 2 diabetes; recent studies have reported antitumor properties across various tumor types (19). Increasing preclinical studies uncovered that metformin impairs the electron transport chain (ETC) and ATP synthesis in cellular metabolism, suppresses oncogenic signaling pathways, inhibits cancer stem cells, and most importantly, improves lymphocyte cytotoxicity, repolarizes macrophages to an anti-tumor phenotype, and reverses the immunosuppressive TME as an immune modulator (20, 21). Metformin has been shown to reprogram the immune microenvironment toward an “infiltrated-inflamed” status by increasing tumor-infiltrating immune-activated cells and decreasing immunosuppressive cells, such as CD4^+^ Treg cells and CD163^+^ macrophages (22). Moreover, metformin has demonstrated growth inhibition across various solid tumors *in vivo*, including ovarian, breast, renal carcinomas, melanoma, non-small-cell lung carcinoma, leukemia, intestinal cancers as well as in gliomas. This effect is largely due to its ability to mitigate CD8^+^ tumor-infiltrating lymphocyte exhaustion and apoptosis, thereby preserving their proliferative capacity and metabolic potential, and to reprogram other immune cell populations within the TME (23, 24). Controversial effects of metformin on high-grade gliomas have also been reported in several clinical trials, showing a potential positive impact of metformin in grade 3 gliomas, whereas no significant benefit in grade 4 gliomas on overall and progression-free survival (25–27). In addition, metformin has also been reported to execute context-dependent role in immunomodulation particularly in benign inflammatory etiology functioning as inflammation attenuator directly working on immune cells which justifies its potential in treating diseases such as neurodegeneration (28, 29). This raised the question of whether metformin also shows context-dependent roles in GBM TME with and without RT and whether tumor-infiltrated immune cells, particularly TAMs in GBM remain plastic enough to react to metformin. These findings highlight metformin as a promising therapeutic agent for glioma treatment; however, optimizing its effectiveness in GBM remains a critical and unresolved challenge.

In this study, we examined the reprogramming effect of metformin on macrophages in multiple tumor models, mimicking an in vitro environment, and evaluated the combined anti-tumor effect of metformin and RT in the GBM mouse model.

## 2 Methods

### 2.1 Cells and cell culture conditions

Bone marrow-derived macrophages (BMDMs) were isolated from femora and tibiae of wild-type B6 mice (12–16 week-old, 20–25 g, Charles River Laboratory). Cells were cultured in Dulbecco’s Modified Eagle Medium (DMEM) supplemented with 10 ng/ml mouse macrophage colony-stimulating factor (m-MCSF, PeproTech, USA), 10% fetal bovine serum (FBS, Gibco, USA), 0.1% penicillin/streptomycin (Gibco, USA), and 1% L-glutamine (Gibco, USA) for 7 days to achieve terminal differentiation into mature BMDMs. In high m-MCSF concentration induction condition, BMDM from wild-type B6 mice (14–16 week-old) were differentiated with DMEM medium that contained 140ng/ml m-MCSF, 10% FBS, 0.1% penicillin/streptomycin, and 1% L-glutamate for 7 days. On day seven, the formation of mature, differentiated BMDMs was evaluated by flow cytometry.

Mouse glioma cell lines CT-2As (with luciferase reporter) and GL261-Luc were obtained from Millipore Sigma. The MGPP3 (PDGF+, P53−/−, PTEN−/−) and PPC2 (PDGF+, P53+/+, PTEN−/−) glioma cell lines were gifted by Dr. Peter Canoll at Columbia University (30). CT-2A and GL261-Luc were maintained in DMEM (Gibco, USA) supplemented with 10% FBS (Gibco, USA) and 1% penicillin/streptomycin (Life Technologies) in a humidified incubator maintained at 37 °C with 5% CO_2_. Cells were passaged using 0.05% trypsin/0.53 mM EDTA. MGPP3 and PPC2 cells were cultured with previously published protocol (30, 31). Only early passages of each cell line were utilized for all experiments.

### 2.2 Cytotoxicity and cell viability assay

BMDM cells, CT-2A, and MGPP3 cells were plated in 96-well plates and treated with increasing concentrations (0-100mM) of metformin. At designated time points (days 1, 3, 5, and 7), cells were washed with phosphate-buffered saline and incubated overnight in metformin-free medium to allow recovery from drug exposure. Cell viability was then determined by the addition of 10μL Cell Titer Blue resazurin reagent (Promega, Madison, WI, USA). Plates were shaken and incubated in the dark for 2 hours, then excited at 560nm and fluorescence measured at 590nm using a Tecan Safire plate reader. All samples were analyzed in triplicate, and standard deviations were calculated. The half-maximal inhibitory concentration (IC50) was determined from dose-response curves using GraphPad Prism software.

### 2.3 Co-culture experiment

In the co-culture experiment, BMDM cells (in the lower chamber) were co-cultured with CT-2A murine GBM cells (Millipore-Sigma, # SCC195) (in the upper chamber) in a 6-well trans-well system. Three conditions were investigated. In the first condition, metformin was added to the medium 24 hours after co-culture of BMDM and CT-2A. In the second condition, BMDM cells pretreated with metformin for 24 hours were then co-cultured with CT-2A for an additional 24 hours. In the third condition, BMDM cells and CT-2A cells co-cultured in a Trans-well system were treated with metformin concurrently for 24 hours. At the end of the treatment, BMDM cells were harvested from the lower chamber, and phenotypic changes were analyzed by flow cytometry. Other glioma cell lines, MGPP3 and PPC2 cells, were gifted by Dr. Peter Canoll at Columbia University Medical Center and were co-cultured with BMDM concurrently with metformin treatment.

### 2.4 RNA sequencing

BMDM cells were treated with metformin (0.1mM and 2mM) for 12 hours and 24 hours. Total RNA was isolated by the RNeasy Plus Mini Kit (Qiagen, Netherlands). The Illumina Universal Plus mRNA-Seq kit was used for preparing RNA-seq libraries. The libraries were sequenced on a single-read, 76-bp output flow cell on the NextSeq 550. For the analysis, each RNA-seq read was trimmed using Trimmomatic to ensure an average quality score greater than 30 and a minimum length of 30 bp (32). All trimmed short reads were mapped to the mouse genome (GRCm38.92) using TopHat, allowing up to two base mismatches per reading (33). Reads mapping to multiple locations were excluded. The number of reads in genes was counted using the HTSeq-count tool with the corresponding mice gene annotations, and the “union” resolution mode was used (34). Relative M1- and M2-related macrophage gene expression across treatment groups was normalized to the control group and expressed as fold change.

### 2.5 Flow cytometry

Flow Cytometry (FC) was used to identify macrophage M1 and M2 phenotypes in all in vitro experiments. Briefly, after incubation under different conditions, BMDMs were collected, and the concentration was adjusted to 1×10^6^ cells/ml in each centrifuge tube. After blocking and labeling with cell viability dye (Thermo Fisher Scientific), BMDM were incubated with F488-conjugated anti-mouse F4/80 (a marker for macrophage), PE-conjugated anti-mouse iNOS (a marker for M1 macrophage), and APC-conjugated anti-mouse CD206 (a marker for M2 macrophage) at 4 °C in the dark for 30 min. The cells were analyzed by BD LSR II. The M1 and M2 macrophages were identified as F4/80^+^iNOS^+^ and F4/80^+^ CD206^+^ cells, respectively.

For multiplex cytokine analysis, the culture medium was collected in all conditions. CCL2, CCL5, CXCL10, CXCL1, and TNF-α tests were performed using the BioLegend Legendplex™ Mouse Anti-Virus Response Panel according to the manufacturer’s guidelines. Flow cytometry was performed using a BD LSR II flow cytometer. Data analysis was performed using LEGENDplex™ software version 8.

### 2.6 Isolation and characterization of immune cells in the mouse brain, spleen, and peripheral blood

For flow cytometric analysis of tumor-infiltrating immune cell populations, mice were euthanized 21 days post-implantation with a lethal anesthetic dose. Mouse brains were removed and gently minced with a clean scalpel, then dissociated in 3ml Accutase solution for 40min at 37 °C with occasional resuspension. Dissociation was stopped by adding 5 mL of prewarmed PBS/10% FBS. Cells were then filtered through a 40-μm cell strainer (BD Falcon), centrifuged at 400g, and resuspended in 4 ml 30% Stock Isotonic Percoll (SIP). The cells were overlaid onto a Percoll gradient (30%/37%/70%) and centrifuged at 800g for 20 min. Lymphocytes were collected from the 37%/70% interphase, washed twice in PBS, then counted and stained with antibodies in conjunction with Mouse BD Fc Block and viability dye.

Spleens were harvested from each mouse, and splenocytes were collected by homogenization of the whole spleen followed by incubation in red blood cell lysis buffer for 5 min. Mice’s peripheral blood was harvested and lysed with red blood cell lysis buffer for 5 min at room temperature. Cells were then washed in PBS and counted. For the flow cytometry experiment, 3 × 10^6^ cells per tube were labeled with the antibodies listed in **Table 1**. Data acquisition was performed on a BD LSR II Green flow cytometer, and subsequent analysis was performed using FlowJo software.

**Table 1:** Antibodies used for flow cytometry.

Antibody	Source	Conjugate	Cat/Clone	Dilution
Anti-mouse CD16/32 (Fc block)	BD Bioscience	N/A	553142	1:50
Anti-mouse CD11b	BioLegend	PE/Cyanine5	101210	1:100
Anti-mouse CD45	BD Bioscience	APC-Cy™7	557659/30-F11	1:100
Anti-mouse F4/80	eBioscience	Alexa Fluor 488	53-4801-82/BM8	1:100
Anti-mouse iNOS	eBioscience	PE	12-5920-82/CXNFT	1:100
Anti-mouse CD206	eBioscience	APC	17-2061-82/MR6F3	1:100
Anti-mouse CD3	BD Bioscience	Brilliant Violet 480	565642/17A2	1:100
Anti-mouse CD4	BioLegend	Brilliant Violet 421	100437/GK1.5	1:25
Anti-mouse CD8	Invitrogen	Super Bright 600	63-0081-82/53-6.7	1:40
Anti-mouse FoxP3	Invitrogen	FITC	MA1-41628/3G3	1:200
Anti-mouse CD44	Invitrogen	PE	12-0441-82/IM7	1:300
Anti-mouse CD62L	Invitrogen	APC	17-0621-82/MEL-14	1:20
Anti-mouse CD11c	eBioscience	BB700	745899/HL3	1:200
Anti-mouse MHCII	BioLegend	Brilliant Violet 421	107632/M5	1:100
Anti-mouse Ly6C	BioLegend	Brilliant Violet 711	128037/HK1.4	1:40
Anti-mouse Ly6G	eBioscience	SuperBright 600	63-5931-82/RB6-8C5	1:300
LIVE/DEAD	Invitrogen	UV Blue	L23105	1:1000

Central memory T cells (Tcm) were identified as CD62L+/CD44+ for CD4 or CD8 lymphocytes, respectively. Effector memory T cells (Tem) were identified as CD62L-/CD44+. mMDSC was identified as CD45^+^/CD11b^+^/CD11c^low^/Ly6C^+^/Ly6G^-/low^/MHCII^low^ and was confirmed to be F4/80 expressing. Granulocytic MDSC (gMDSC) or PMN-MDSC was identified as CD45^+^/CD11b^+^/CD11c^low^/Ly6G^high^. Macrophages and microglia in brain tumor and non-tumor tissues were distinguished by CD45 and CD11b expression after live-cell gating. Infiltrating macrophages were defined as CD45^hi^ CD11b^+^ cells, whereas resident microglia were defined as CD45^int^ CD11b^+^ cells. Lymphocytes were defined as CD45^hi^ CD11b^-^ cells. iNOS and CD206 expression were analyzed within the CD45^hi^ CD11b^+^ macrophage gate to evaluate M1-like and M2-like macrophage features.

### 2.7 Mouse models and treatment

Nine- to ten-week-old female Albino B6 mice were purchased from Jackson Laboratory and were housed in pathogen-free conditions at the University of Nebraska Medical Center (UNMC) until 12 to 13 weeks of age. All experimental procedures were performed in accordance with a protocol approved by the University of Nebraska Medical Center, Institutional Animal Care, and Use Committee.

To establish a murine glioma model, dissociated CT-2A cells or GL261-Luc (5 × 10^4^ cells suspended in 2 μl PBS) were injected stereotactically into the right caudate-putamen (0.1 mm anterior and 2.25 mm lateral to bregma and 2mm deep) of the female albino B6 mice (age 12–13 weeks). Seven days after tumor implantation, mice were imaged using the IVIS platform to monitor tumor growth and then stratified into experimental treatment groups based on luminescence, such that the average tumor radiance in each group was statistically comparable. The tumor take rate was 100%. The treatment groups were as follows: control (saline administered intraperitoneally), Metformin, RT (saline administered intraperitoneally), and Metformin+RT. Mice in the metformin treatment group received an intraperitoneal (*i.p.*) injection of metformin (Sigma, US) at 250mg/kg daily from day 8 to day 28 after tumor inoculation. Mice in the RT group were also irradiated with 8 Gy on day 11 using the RS-2000 irradiator. Specifically, mice were anesthetized and irradiated with their bodies covered by a lead shield. On day 20, after inoculation, blood samples from the tail vein were collected from 3 mice per group for blood glucose testing using the Contour Next (Bayer, US). Day 21 after tumor implantation, 5–6 mice from each group were sacrificed, and the brain tissues were harvested for Hemotoxylin and Eosin (H&E) or for immune cells detection by immunohistochemistry (IHC) or flow cytometry. Spleen and peripheral blood were also collected for immune cells profiling by flow cytometry. The rest of the mice were followed to record their survival, body weight, and tumor growth following endpoint protocols outlined in the approved animal protocols and were euthanized when they displayed predetermined signs of neurologic deficits. For the GL261 glioma model, Metformin (500 mg/kg in 100 μL of PBS) was administered by oral gavage daily starting on day 8 after tumor inoculation for 5 weeks or until the endpoint. Mice in the RT and dual-treatment groups received RT (8 Gy) on day 11. Mouse body weight and tumor growth were monitored weekly until the endpoint.

### 2.8 *In vivo* bioluminescence analysis

To monitor tumor growth in living animals, all CT-2A and GL261-Luc cells used for animal studies were stably transduced with a firefly luciferase-EGFP reporter. To examine tumor growth, mouse brains implanted with CT-2A or GL261 cells were monitored weekly using bioluminescent imaging. Animals were injected intraperitoneally with 120mg/kg body weight of d-luciferin and anesthetized with isoflurane for imaging analysis. The tumor luciferase images were acquired using IVIS (*In Vivo* Imaging System; Caliper Life Sciences).

### 2.9 Immunohistochemistry

After DPBS perfusion, the mice brains were collected, fixed in 10% buffered formalin for 24h, then transferred to 70% ethanol, embedded in paraffin, and sectioned by the Tissue Core Facility at UNMC. The paraffin sections were cut into 4 μm sections. Hematoxylin and eosin (H&E) staining were performed. Images were acquired at ×200 and ×400 magnification using a standard light microscope. The blank slides were incubated with anti-iNOS (ab15323, Abcam), anti-CD206 (ab64693, Abcam), anti-CD3 (MA1-90582, Invitrogen), anti-CD4 (ab183685, Abcam), anti-CD8 (ab209775, Abcam), and FoxP3 (ab215206, Abcam). Scans were aligned with Ventana iScan HT slide scanner and analyzed with Definiens Tissue Studio (Ventana, Munich, Germany).

### 2.10 Statistical analysis

All data were analyzed using GraphPad Prism 9.0 (GraphPad, USA). Results are expressed as mean ± standard deviation or standard error of the mean. One way ANOVA and Tukey multiple comparison test were used to calculate the significant difference between multiple groups. Kaplan-Meier survival curves were analyzed using the log-rank (Mantel-Cox) test. *P* values <0.05 were considered statistically significant. Statistical notation: *:*P* < 0.05; **: P < 0.01; ***: *P* < 0.001; ****: P < 0.0001; n.s.: not significant.

## 3 Results

### 3.1 Metformin-induced cytotoxicity in GBM tumor cells requires much higher drug concentration (mM) than that for modulation of macrophage homeostasis/polarization (µM)

**Figure 1:**
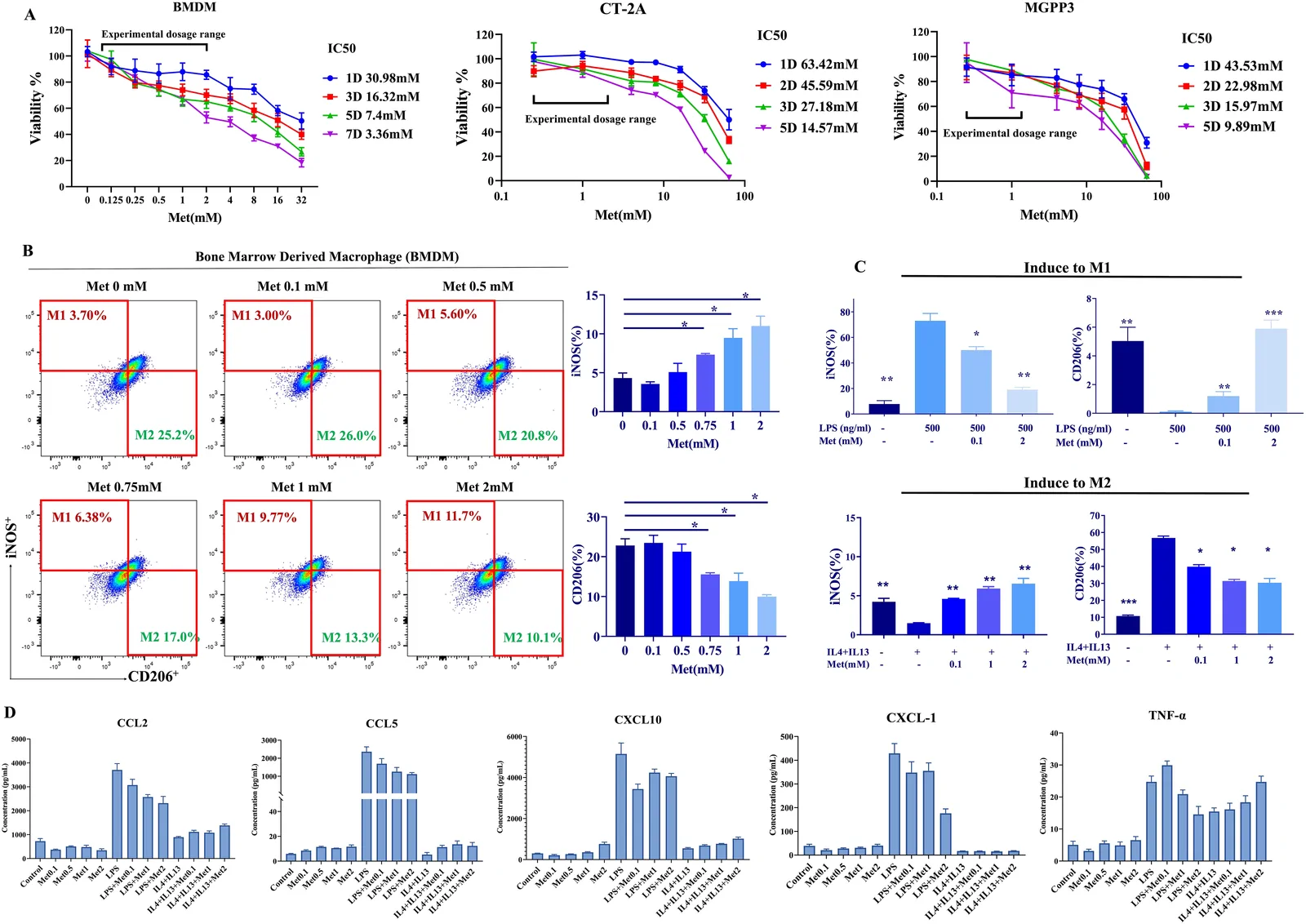
The dual regulation of metformin in macrophage differentiation. **(A)** Cell viability of BMDM, CT-2A, and MGPP3 cells were assessed using the CellTiter-Blue assay following treatment with increasing concentrations of metformin for 1, 3, 5, and 7 days. Dose–response curves were used to calculate IC50 values at each indicated time point. Data are presented as mean ± SD from three independent experiments, each performed in triplicate. **(B)** Metformin stimulates mouse bone marrow derived macrophage (BMDM) into an anti-tumor phenotype. Flow cytometry analysis of iNOS (M1 marker) and CD206 (M2 marker) was performed after treatment with increasing concentrations of metformin (0, 0.1, 0.5, 0.75, 1, and 2 mM) for 24 h. Representative dot plots show CD206^-^ iNOSα (M1) and CD206α iNOS^-^ (M2) populations. Bar graphs represent the percentage of CD206^-^ iNOSα and CD206α iNOS^-^ BMDMs. *P < 0.05 versus the 0 mM metformin control; n = 3. **(C)** BMDM cells were first incubated with LPS (500ng/ml) or IL-4 (10ng/ml) combined with IL-13 (10ng/ml) for 24h to induce M1 or M2 macrophage. Subsequently, LPS or IL-4 + IL-13 were removed, and BMDMs were treated with varying concentrations of metformin for 24 h. The M1 (iNOS^+^)/M2 (CD206^+^) phenotype change of BMDM was analyzed by flow cytometry. *p <0.05, **p ≤ 0.01, ***p ≤ 0.001 vs. the LPS or IL-4+IL-13 only group; n=3. **(D)** Expression levels of anti-tumor cytokines. Culture medium was collected from experiments in **(B, C)**, and cytokines released in each condition were detected.

The Cell Titer-Blue assay was used to evaluate the effect of metformin on the growth of mouse bone marrow-derived macrophages (BMDMs) and two murine GBM cell lines. BMDM cells, harvested from Albino B6 mice at 12–14 weeks of age, were treated with increasing concentrations of metformin for 1day, 3 days, 5 days, and 7 days. Murine glioma cells CT-2A and MGPP3 were treated with metformin for 1, 2, 3, and 5 days. As shown in **Figure 1A**, the IC50 values for BMDM cells decreased progressively from 30.98 mM at 1 day to 16.32 mM at 3 days, 7.4 mM at 5 days, and 3.36 mM at 7 days of metformin exposure, indicating cumulative toxicity over time. For CT-2A glioma cells, metformin exhibited IC50 values of 63.42 mM at 1 day, 45.59 mM at 2 days, 27.18 mM at 3 days, and 14.57 mM at 5 days. MGPP3 cells demonstrated greater sensitivity to metformin compared to CT-2A, with IC50 values of 43.53 mM at 1 day, 22.98 mM at 2 days, 15.97 mM at 3 days, and 9.89 mM at 5 days. A 10 mM metformin dose decreased the MGPP3 cell growth by approximately 30% and 50% after 3 days and 5 days of treatment, respectively. Metformin induced insignificant inhibition on cell proliferation at doses up to 2 mM after 1 day and less than 50% even after 7 days of treatment on BMDM. After 3 days of treatment, the same dose resulted in less than 10% suppression in CT-2A and MGPP3 cells. This safe window was used to conduct further experiments to test the modulation effect of metformin on BMDM differentiation under conditions mimicking the GBM environment.

Further, we examined the effect of metformin on macrophage (BMDM) differentiation *in vitro*. Metformin significantly promoted M1 macrophage (M1*Φ*) while downregulated M2 macrophage (M2*Φ*) most effectively in the 1mM and 2mM range but starting at high micromolar levels (**Figure 1B**). Consistently, metformin partially reversed the M2*Φ* -induction and M1*Φ*-suppression by IL-4 and IL-13 combination treatment at as low as 0.1mM concentration (**Figure 1C**). IL-4/IL-13 were reported to induce M2*Φ* in both benign and cancer conditions (35–37). However, when pretreated BMDM with a potent inflammatory inducer, lipopolysaccharide (LPS), metformin, rather than further increase M1*Φ*, decreased the pro-inflammatory effect of LPS and restored M2 starting at 0.1mM. The effects of LPS on M1*Φ* and M2*Φ* were completely restored by metformin at 2mM (**Figure 1C**). Anti-tumor cytokines/chemokines, CCL5, CXCL10, and TNF-α secreted by M1*Φ* and pro-tumor CCL2 and CXCL1 were tested. Metformin increased the level of M1*Φ*-related cytokines/chemokines at a dose-dependent manner (**Figure 1D**). LPS markedly increased the secretion of cytokines and chemokines by M1*Φ*, whereas metformin partially attenuated this effect. Similarly, pretreatment with IL-4 and IL-13 decreased (CXCL-1) or had no effects (CCL2, CCL5 and CXCL-10) on the levels of M1*Φ*-related cytokines/chemokines, whereas metformin reversed and/or increased their levels (**Figure 1D**).

### 3.2 Metformin promotes macrophage phenotype switching towards an anti-tumor phenotype from pre-induced M2 phenotype

**Figure 2:**
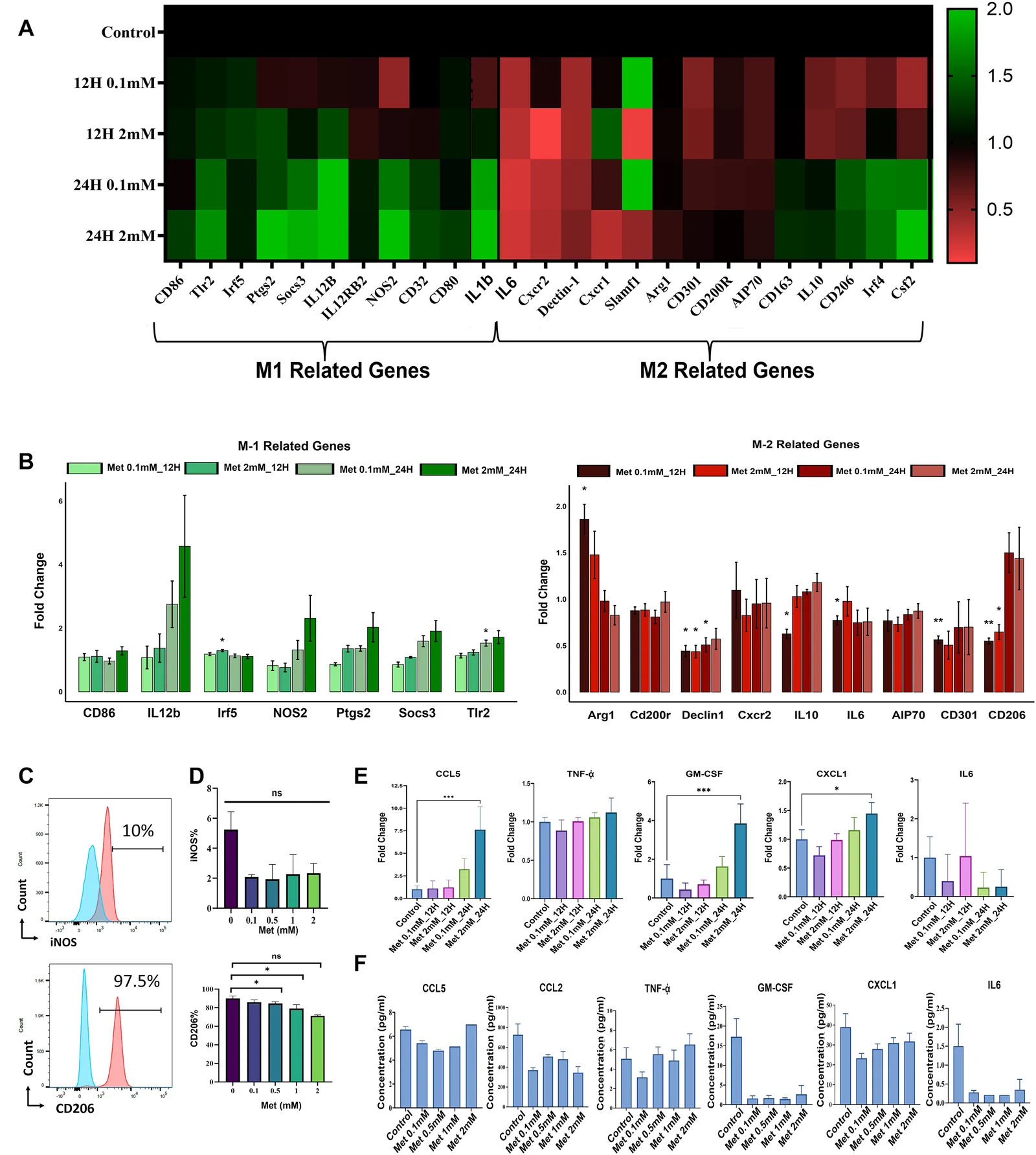
Transcriptional profiles of BMDM treated with metformin. BMDMs were harvested from male B6 Albino Mice and treated with metformin (0.1 mM and 2 mM) for 12 and 24 h. After treatment, BMDM was washed, and RNA was extracted for RNA-seq analysis. Data are representative of three independent experiments. **(A)** Heatmap showing the fold change of M1 macrophage and M2 macrophage related genes after treated with metformin (0.1mM and 2mM) for 12h and 24h. Data were normalized to the control group. **(B)** Relative fold changes of M1 and M2 macrophage-related gene levels were normalized with the gene levels in the control group. **(C)** Flow cytometric analysis confirming M2 polarization with minimal iNOS expression (10%) and robust CD206 expression (97.5%), indicating a CD206-dominant macrophage population for metformin treatment studies. **(D)** Quantification of iNOS, and CD206 percentage after metformin treatment (0-2mM). **(E)** The RNA-sequencing analysis further demonstrates the time and dose-dependent effects of metformin on the mRNA expression of cytokines in the BMDM. The mRNA levels were calculated as linear fold change normalized to the mean of the control. Significant upregulation in the level of CCL5, GM-CSF, and CXCL1 was observed at 24h, whereas TNF-α, and IL-6 level didn’t change significantly (*p < 0.05, and ***p < 0.001 vs. Control. **(F)** The pro-inflammatory cytokines in cultured media conditioned by BMDM for 24 hours were measured by BioLegend LEGENDplex panel, which is a bead-based flow cytometry immunoassay. The controlmacrophages showed minimal M1*Φ* cytokine expression. M2*Φ*-associated cytokines such as CXCL1, IL-6 and GM-CSF were significantly downregulated by metformin treatment. Overall, in pre-induced M2*Φ* condition, metformin showed significant suppressive effects on M2*Φ* phenotype but likely an incomplete subtype switching to functional M1*Φ*.

To evaluate the immunomodulatory effects of metformin more specifically in tumor-associated macrophage (TAM) in GBM that are predominantly immunosuppressive subtype, mouse BMDMs were differentiated with a high concentration of Macrophage Colony-Stimulating Factor (M-CSF) (140 ng/mL) for one week to generate a predominant M2 phenotype mimicking TAM (**Figure 2**) (38, 39). Flow cytometric analysis confirmed successful polarization, with 97.5% of cells expressing the M2*Φ* marker CD206 (**Figures 2C, D**, lower panel). In contrast, only 10% of the cells expressed the M1*Φ* marker iNOS, indicating minimal pro-inflammatory activation (**Figures 2C, D**, upper panel). This immunophenotype, characterized by high CD206 and low iNOS levels, as well as high IL10/IL12 ratios of expression after induction, establishes a robust M2-predominant population that is well suited for assessing metformin’s modulation of the M1/M2 macrophage balance within an immunosuppressive microenvironment mimicking tumor microenvironment.

To verify whether metformin promotes an anti-tumor phenotype in macrophages in the pre-induced M2 phenotype, we performed RNA sequencing (RNA-seq) analysis to study the transcriptomic signatures of mouse BMDM cells after treatment with low- (0.1mM) and high-dose (2mM) metformin for 12 and 24 hours (**Figure 2A**). The 12-hour metformin treatment produced modest increases in M1-related gene expression at 0.1 mM, whereas 2 mM metformin elicited substantially greater induction of multiple M1*Φ* signature genes, including *CD86*, *Tlr2*, *Ptgs2*, *Socs3*, *IL-12b*, and iNOS (*NOS2*). The effects were also time-dependent (**Figure 2B**, left panel). At 12 hours, metformin treatment downregulated M2 signature genes, including *IL-6*, *CD301*, *CD206r*, *Dectin1*, *AIP70*, *Cxcr2*, *CD206*, and *IL*-10 at both 0.1 mM and 2 mM concentrations, without a clear evidence of dose-dependency. Extension to 24 hours of treatment produced divergent responses within the M2 gene set: certain genes, such as IL-6 and Dectin1, maintained sustained downregulation relative to untreated controls, whereas others, including CD301 (Clec10a), CD206 and IL-10, exhibited partial recovery or reversal toward baseline expression levels (**Figure 2B**, right panel). These findings indicate that metformin exerts time- and dose-dependent promotion of M1*Φ* -related genes, with maximal effect at the tested 2 mM dose, concurrently with pronounced but temporally more dynamic suppression of M2-related genes, which shows greater inhibition at 12 hours than at 24 hours.

To evaluate whether the above observed changes at gene expression level promoted by metformin specifically those chemokines can indeed translate to functional M1*Φ* polarization from a heavily M2*Φ* polarized subtype, we measured pro-inflammatory cytokines in cultured media conditioned by BMDM for 24 hours. Overall, control macrophages showed minimal M1*Φ* cytokine expression. A few detectable cytokines including TNF-α, CCL5 and CCL2 were not increased after 24 hours of metformin treatment despite some showing increased mRNA expression, *e.g.*, CCL5 (**Figure 2F**). Other M1*Φ* -associated cytokines such as IFN-γ, and IL-12 was not detectable (data not shown). In contrast, M2*Φ*-associated cytokines such as IL-6, and GM-CSF were significantly downregulated by metformin treatment, and the effects were not dependent on drug concentration at the tested range from 0.1 mM to 2 mM. Overall, in pre-induced M2*Φ* condition, metformin showed significant suppressive effects on M2*Φ* phenotype but likely an incomplete subtype switching to functional M1*Φ*. The RNA-sequencing analysis further demonstrates the fold change in these pro-inflammatory cytokine levels after 0.1mM, and 2mM metformin treatment for 12h and 24h (**Figure 2E**). This incomplete functional M1*Φ* promotion by metformin is consistent with flow cytometry data showing no significant increase but rather decrease in iNOS+ cells as well as only mildly decreased CD206+ cells after metformin treatment in pre-induced M2*Φ* (**Figures 2C, D**).

Further, we did Gene Set Enrichment Analysis (GSEA) in BMDM cells after 0.1mM, and 2mM metformin treatment for 12h and 24h (**Supplementary Figures 1A–C**). The GSEA results based on Hallmark pathway database showed that pro-inflammatory effect is upregulated after high dose of metformin treatment; functionally homeostasis promotion is consistently ranked among the top pathways after treatment (Reactome database). Taking into consideration together with the upregulation in IL2, and IL6 Jak Stat3 signaling pathways in prolonged drug treatment (24h), our data strongly indicates metformin functions through promoting homeostasis rather than simply switching BMDM M1-type macrophage. Heatmaps were generated based on RNAseq results on representative genes that confirmed the promoting effects of metformin on the homeostasis pathway (**Supplementary Figure 1D**).

### 3.3 Modulation of macrophage polarization by metformin in the conditions mimicking GBM microenvironment *in vitro*

**Figure 3:**
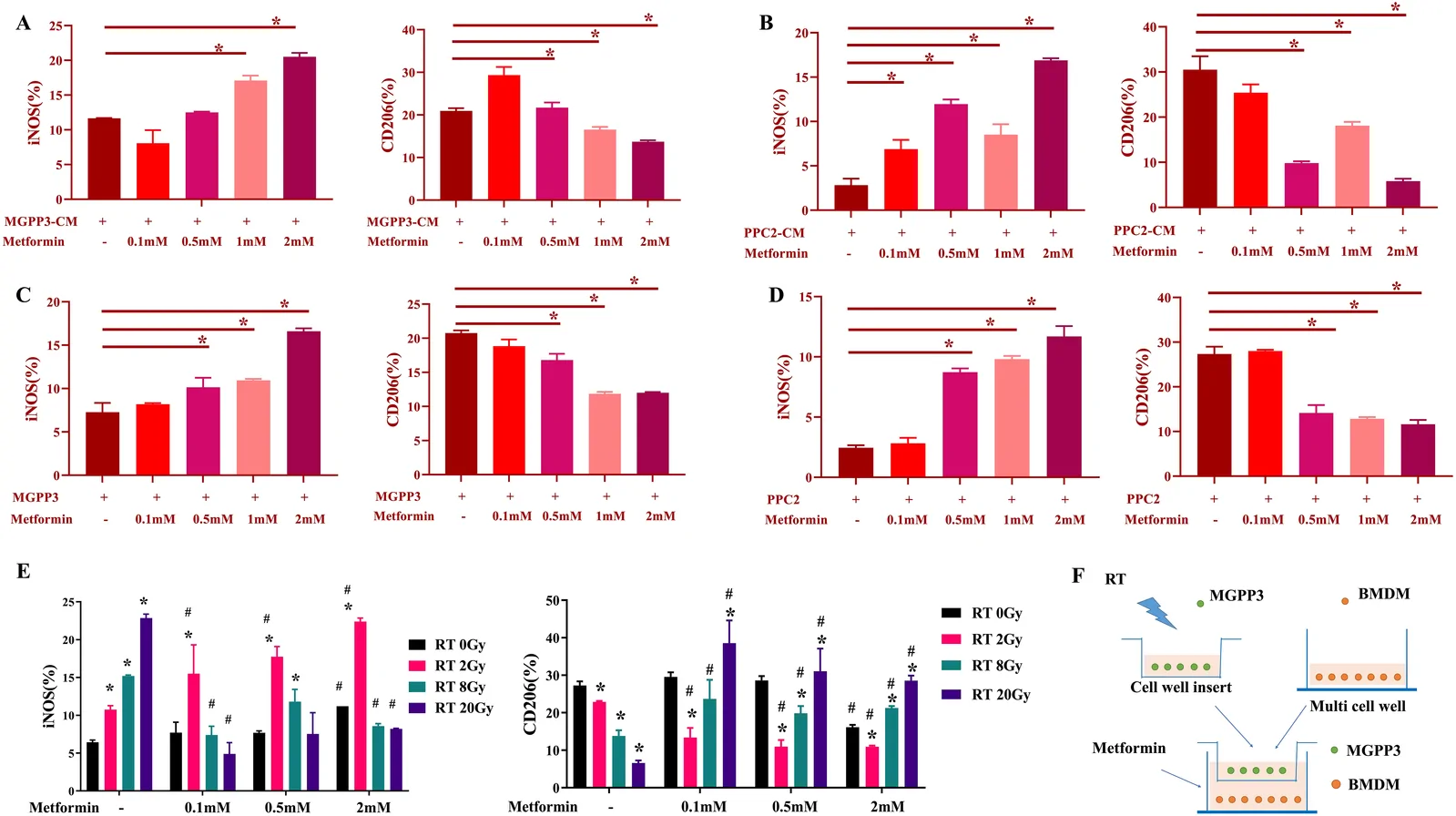
Metformin reprograms macrophage polarization in conditions mimicking tumor environment. **(A, B)** Metformin-treated BMDMs cultured with GBM tumor-conditioned medium increase M1-associated marker expression and reduce M2-associated marker expression. Conditioned medium from GBM tumor cells was harvested from MGPP3 **(A)** or PPC2 **(B)** cells after 24 h of culture (MGPP3-CM and PPC2-CM). Incubated BMDM with 20% condition medium collected from GBM cells with or without a different concentration of metformin for 24h. The M1 (F4/80^+^iNOS^+^ CD206^-^)/M2 (F4/80^+^iNOS^-^ CD206^+^) phenotype change of BMDM was analyzed by flow cytometry. *P < 0.05 comparison to no metformin treatment group; n=3. **(C, D)** BMDM were seeded in 6-well plates, and MGPP3 **(C)** or PPC2 **(D)** cells were seeded into Falcon 6-well cell culture inserts. Both the BMDM and GBM cells were cultured separately overnight, then co-cultured for 24 h. The M1 (iNOS^+^)/M2 (CD206^+^) marker expression change of BMDM was analyzed by flow cytometry. *p < 0.05 vs. the no metformin treatment group; Values are expressed as the mean ± SE of three experiments. **(E)** Phenotype change of BMDM co-cultured with MGPP3 pre-treated with RT. MGPP3 cells were cultured in 6-well cell culture inserts. When the adherent cells reached 70% confluence, they were changed with fresh medium and irradiated the MGPP3 cells with 0Gy, 2Gy, 8Gy, and 20 Gy doses. Immediately after the RT, place the inserts containing MGPP3 into the 6-well cell culture plates containing BMDM and co-culture for 24 h. Harvested the BMDM and analyzed the M1 (iNOS^+^)/M2 (CD206^+^) phenotype change by flow cytometry. *p < 0.05 vs. no RT (RT 0Gy) treatment group in the same concentration condition of metformin treatment; ^#^p < 0.05 vs. no metformin treatment group in the same dose of RT condition. Values are expressed as the mean ± SE of three experiments. **(F)** Diagram illustrated co-culture of MGPP3 with BMDM.

To evaluate the immunomodulatory impact of metformin on macrophage phenotype within the glioblastoma (GBM) microenvironment, BMDMs were subjected to glioma-conditioned medium (CM) as well as trans-well co-culture with murine glioma cell lines. **Figure 3** summarizes the results of mouse glioma cells interacting with BMDM with and without metformin treatment in various sequencing.

MGPP3 CM and PPC2 CM were harvested from the supernatant of MGPP3 cells and PPC2 cells’ culture medium after 72 hours of culture. MGPP3 cells, after being implanted to the mouse brain, behave like a high-grade glioma (HGG) or GBM; while PPC2 is consistent with a low-grade glioma (LGG) (30, 31). BMDM was treated with 20% CM along with metformin (0.1mM, 0.5mM, 1mM, and 2mM) for 24 hours. Changes in BMDM phenotype were assessed by flow cytometry using the markers F4/80^+^/iNOS^+^/CD206^-^ (M1*Φ*) and F4/80^+^/iNOS^-^/CD206^+^ (M2*Φ*). Compared with the CM-only group, a significant dose-dependent increase in iNOS-positive cells and a decrease in CD206-positive cells were observed when BMDM cells were treated with metformin at concentrations above 1 mM in the MGPP3 CM environment (**Figure 3A**) but at much lower concentration, *i.e.*, as low as 0.1 mM in the PPC2 CM environment (**Figure 3B**). Metformin at 2mM decreased about 50% M2 macrophage in MGPP3 CM and more than 90% in PPC2 CM condition. We further examined the effect of metformin in the co-culture system of GBM and BMDM cells (**Figures 3C, D**). MGPP3 or PPC2 glioma cells and BMDM cells were co-cultured in a Transwell system with/without metformin for 24 hours. BMDM cells were collected from the lower chamber, and the phenotype changes were analyzed by flow cytometry. Consistent with the CM system, metformin at 0.5mM to 2mM range could significantly switch macrophages to the M1 phenotype and inhibit the differentiation of the M2*Φ* phenotype when co-cultured with MGPP3 cells (HGG) with metformin’s effects to switch the phenotype being much more prominently when co-cultured with PPC2 (LGG). Together, these data suggested that metformin could effectively promote the polarization from M2*Φ* to M1*Φ* subtype in conditions mimicking GBM microenvironment but less effective when exposed to more aggressive type of glioma cells.

### 3.4 Combination effect of metformin and radiotherapy on macrophages in GBM environment

As the cornerstone of treatment for GBM, radiotherapy (RT) induced inflammation is a key contributor to cancer eradication but also causes injury to the brain tissue with the RT field due to inflammation induced by RT including upregulation of the secretion of pro-inflammatory cytokines and the stimulation of M1*Φ*. Therefore, we next explored the effect of metformin on macrophages in combination with RT in *in vitro* conditions mimicking GBM microenvironment. RT Doses ranging from 2Gy to 20Gy were used to irradiate MGPP3 cells. Immediately after the RT, MGPP3 cells were co-cultured with BMDM for 24 hours (**Figure 3E**). The changes in the markers for M1*Φ* (F4/80^+^iNOS^+^CD206^-^) and M2*Φ* (F4/80^+^ iNOS-CD206^+^) macrophage were determined by flow cytometry. As shown in **Figure 3E**, RT alone significantly increased M1*Φ* and inhibited M2*Φ* polarization of macrophages in a dose-dependent manner. Metformin further enhanced the pro-inflammatory effects of low-dose (2Gy) RT, whereas it attenuated the M1*Φ*-promoting effects of moderate- and high-dose RT (8Gy and 20Gy). Notably, effective concentrations of metformin are as low as the tested 0.1mM. Increasing metformin concentrations (0.5 mM and 2 mM) did not further reverse the M1-promoting or M2-inhibiting effects of RT at 8Gy or 20Gy, but maintained the M1/M2 balance at the baseline/control level without RT. These data suggested that metformin functioned likely as a modulator instead of a one-way promoter of M1*Φ*, *i.e.*, metformin enhanced pro-inflammatory effects at low level of inflammation induced by low dose RT but could neutralize high dose RT-induced high level of inflammation and M1*Φ* promotion in a GBM environment.

### 3.5 The combination of metformin and RT effectively suppresses tumor growth *in vivo*

**Figure 4:**
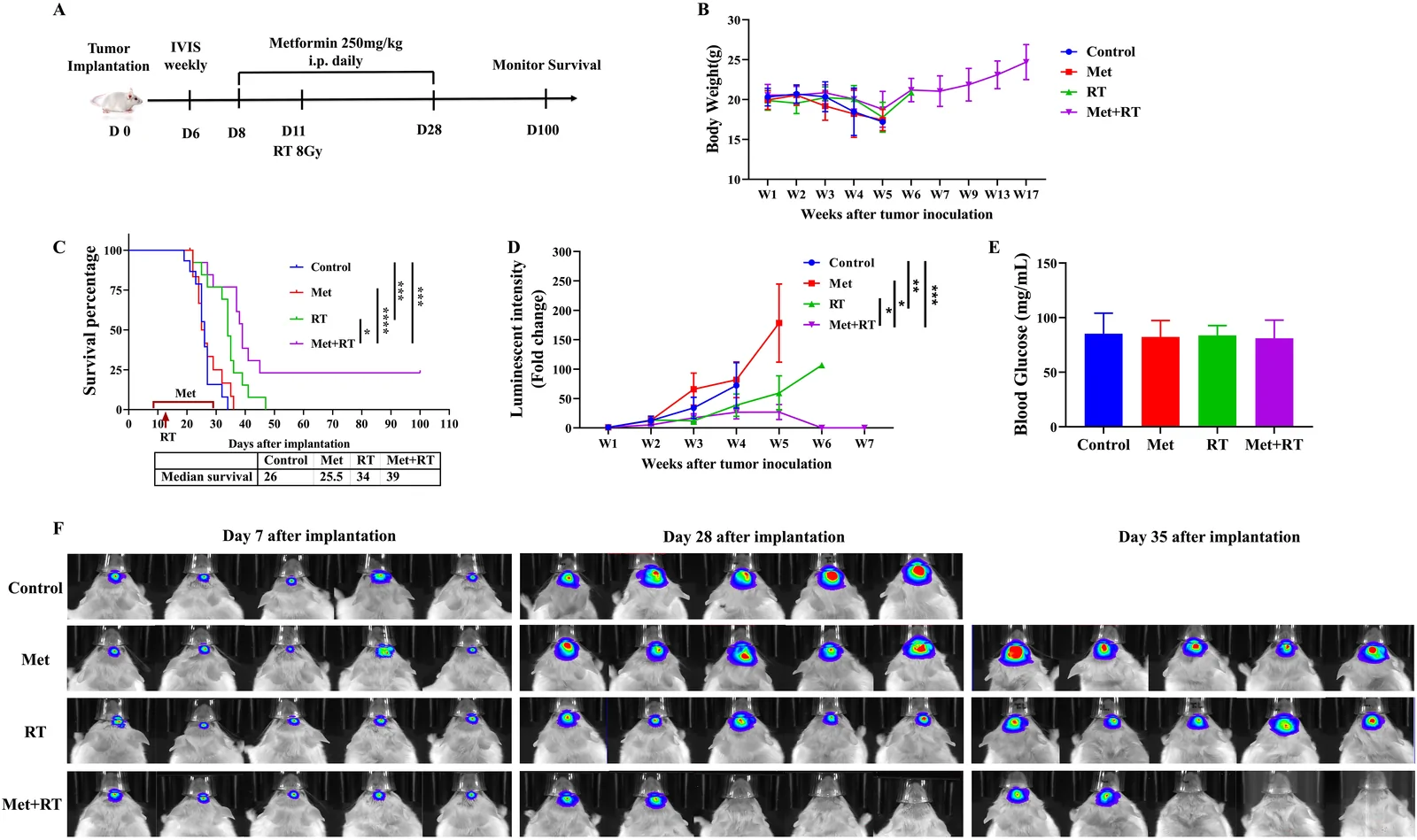
The combination of metformin and RT suppresses tumor growth *in vivo*. **(A)** Time course of tumor implantation and metformin and RT administration. CT-2A murine glioma cells were stereotactically implanted into the right caudate and putamen of 12-week-old female C57BL/6 mice that were treated intraperitoneally with saline or 250 mg/kg metformin daily for 3 weeks. Selected groups received RT (8 Gy) as monotherapy or combination therapy on day 11. **(B)** After tumor implantation, body weight was recorded from week 1 to week 17. No significant difference between groups was found. **(C)** Kaplan–Meier survival analysis of control, metformin (Met), RT, and Met+RT–treated mice (n = 15 per group at study initiation). Statistical differences between the Met+RT group and the other groups were analyzed using the log-rank (Mantel-Cox) test; *p < 0.05, ***p < 0.001, ****p < 0.0001. **(D)** IVIS bioluminescence imaging was performed weekly to monitor tumor burden. The control and metformin-alone groups exhibited progressive increases in tumor signal, whereas the RT and Met+RT groups showed marked suppression of bioluminescence. Quantification represents photon flux (photons/sec). Data are shown as mean ± SD (n = 13 mice per group). *p < 0.05; **p < 0.01; ***p < 0.001. **(E)** Blood glucose was measured on day 12 following metformin treatment. No statistically significant difference was found among the four groups. Data represents SD, n=3. **(F)** Representative IVIS images of tumor-bearing mice on days 7, 28, and 35 following tumor implantations.

To investigate the potential of metformin as a combinatorial therapeutic approach with radiotherapy (RT) in a clinically relevant setting, various treatment regimens were assessed in orthotopic CT-2A-derived glioma models established in immunocompetent Albino B6 mice (**Figure 4A**). The experimental timeline indicates that tumor implantation occurred on Day 0, followed by IVIS imaging on Day 6 to verify tumor establishment. Metformin (250 mg/kg) was administered via intraperitoneal injection (*i.p.*) daily for 21 consecutive days, commencing on the eighth day post-tumor cell implantation. Mice in the Met+RT and RT groups received 8 Gy radiation to the whole brain on Day 11 as monotherapy or combination therapy. Body weight and blood glucose levels were monitored weekly to evaluate potential toxicity. No survival advantage was observed with metformin monotherapy compared to the control group (median survival, 26 days; P = 0.45). In contrast, RT monotherapy (8 Gy single dose) significantly enhanced overall survival relative to the control group (P = 0.005), extending median survival to 34 days, which represents a 31% improvement. However, the combination of RT and metformin conferred the most substantial survival benefit. The Met+RT treatment achieved a median survival of 39 days, which was significantly superior to both metformin monotherapy (P<0.001) and RT monotherapy (P = 0.02). Notably, 3 out of 15 mice (20%) in the Met+RT group achieved long-term survival, remaining alive beyond 100 days post-tumor implantation without evidence of tumor recurrence or gross neurological impairment. These three cured mice exhibited complete tumor clearance by Day 28 post-implantation, corresponding to approximately 3 weeks post-implantation, 13 days after the initiation of metformin treatment, and 10 days after RT administration (**Figure 4C**). Furthermore, no differences in body weight or blood glucose levels were observed among the four groups suggesting good tolerance of mice to metformin or Met+RT (**Figures 4B, E**), suggesting the absence of significant toxicity or hypoglycemia in the metformin-treated groups. Collectively, the combination of metformin and RT demonstrated significant improvements in tumor burden and survival, with no notable alterations in body weight or blood glucose.

Tumor growth patterns, assessed *via* luciferase imaging (IVIS), corroborated the survival data. IVIS imaging revealed rapid tumor progression in the control and metformin groups. In the RT monotherapy group, tumor growth was halted immediately following irradiation but resumed one week thereafter, resulting in the demise of all mice within 7 weeks. Mice receiving Met+RT exhibited significantly slower tumor progression after Week 3 compared to the RT monotherapy (P = 0.02) and metformin monotherapy (P = 0.03) groups. Met+RT-treated mice maintained substantially lower bioluminescence values throughout the study duration, with luminescence remaining below 150 p/s even by Week 4, indicative of sustained tumor growth suppression (**Figure 4D**). IVIS imaging on Days 28 and 35 post-implantations confirmed these quantitative observations, with Met+RT-treated mice showing minimal bioluminescent signals in comparison to the intense signals in the control, metformin, and RT monotherapy groups, and a reduction in tumor burden exceeding 50% relative to the other treatment groups (**Figure 4F**).

To further substantiate the hypothesis that metformin serves as an effective combinatorial strategy with RT in GBM, another established GBM mouse model, the GL261 orthotopic GBM model, was selected to examine the combined effects of metformin and RT. Although both the CT-2A and GL261 models are syngeneic models, thereby more accurately recapitulating the immune response in human GBM, GL261 model is known to be less immunosuppressive in TME as CT-2A model. Placebo (saline) and metformin (500 mg/kg/d) were administered daily *via* oral gavage to GL261 tumor-bearing mice for five weeks or until endpoint. Consistent with observations in the CT-2A model, metformin treatment augmented the therapeutic efficacy of RT by reducing tumor volume and significantly prolonging median survival to 77.5 days, compared to 28 days in the control group and 37 days in the RT monotherapy group. Five out of 13 mice in the Met+RT treatment group were cured and achieved long-term survival following a 100-day follow-up period (**Supplementary Figures 2A–C**).

### 3.6 Metformin reverses immunosuppressive tumor microenvironment but neutralizes the RT-induced inflammation in tumor-bearing mice

**Figure 5:**
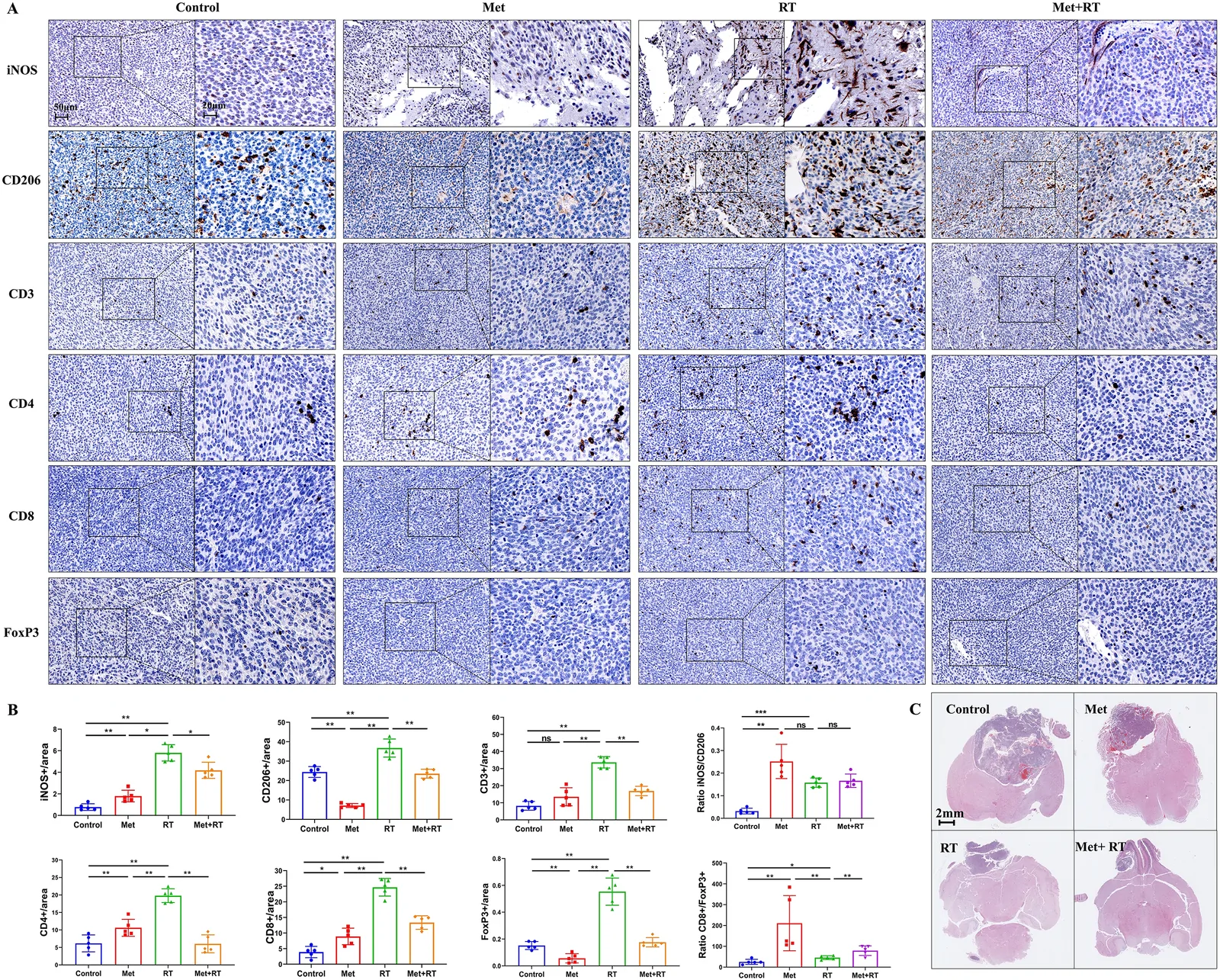
Immune cell infiltration within the intracranial tumor following metformin combined with RT. C57BL/6 mice bearing intracranial CT-2A tumors were treated with saline, metformin, RT, or Met+RT (n = 15 mice per group at study initiation). Brains were harvested for histopathology and immunohistochemistry when the mice were terminally ill from tumor burden (survival endpoint). **(A)** Representative immunohistochemical staining of intracranial tumor sections for iNOS (M1-associated macrophage marker), CD206 (M2-associated macrophage marker), CD3, CD4, CD8, and FoxP3 (regulatory T cell marker). Images show low-magnification views (200×, scale bars = 50 μm) and corresponding high-magnification insets (400×, scale bars = 20 μm). **(B)** Bar graphs represent the total number of positive cells per mm^2^ tumor tissue for iNOS, CD206, CD3, CD4, CD8, FoxP3, M_1_/M_2_ ratio, and CD8^+^/FoxP3^+^ ratio in control, Met, RT, and Met+RT groups; Bars represent mean ± SEM. Each symbol corresponds to one mouse, n=5; *p <0.05, **p < 0.01, ***p < 0.001, n.s., not significant. **(C)** Representative images of H&E-stained cross sections of tumor-bearing brains from mice in control, Met, RT, and Met+RT groups (black scale bar =2mm).

To further evaluate changes in tumor-infiltrating immune cells in GBM-bearing mice, we first performed immunohistochemistry (IHC) assays with multiple lymphocyte and macrophage markers. As anticipated, RT alone significantly increased infiltration by most immune cells in the tumor area; in particular, there was an increased frequency of iNOS+ M1 macrophages and a higher iNOS/CD206 ratio compared with the control group. After RT, the frequency of FoxP3+ cells, a marker of T regulatory cells, in brain tumors also increased considerably, potentially reflecting a complex response to brain RT that may induce immunosuppressive TME and poor clinical outcome (40). Metformin alone robustly increased the iNOS/CD206 and CD8/FoxP3 ratios by 2.3-fold and 7.9-fold compared with the control group (P<0.01), indicating a shift toward a more anti-tumor immune environment. When combined with RT, metformin modulated the pro-inflammatory environment induced by RT by decreasing the proportion of iNOS and simultaneously decreasing Treg, leading to an increase in the CD8+/FoxP3 ratio of 1.7-fold compared with RT alone, suggesting that metformin aids in relieving immunosuppressive TME as an overall effects but may safeguard the response to RT to prevent overstimulation (**Figure 5B**). Representative mouse brain/tumor specimens are shown in **Figure 5A**. The H&E staining of cross-sections of the dissected mouse brains showed hemorrhage and necrosis in the control and the metformin alone groups. The tumor volume significantly declined after Met+RT compared with the control, Met alone, or RT alone treated mice (**Figure 5C**).

We further examined the immune response to metformin and RT treatment in non-tumor brain regions (contralateral side of hemisphere to the injection site) of CT-2A tumor-bearing mice. As shown in **Supplementary Figure 3**, RT alone does not affect the iNOS-to-CD206 ratio but significantly increases the CD8^+^ T cell-to-regulatory T cell ratio in the non-tumor area, which is opposite to the tumor area. Metformin alone and dual treatment increase the iNOS/CD206 and CD8^+^/FoxP3^+^ ratios, indicating that metformin regulates the immune environment across the whole brain by promoting M1 macrophage polarization and enhancing T-cell infiltration, particularly CD8^+^ T cells, but by downregulating FoxP3^+^ cells or Treg only in tumor environment but not in normal brain tissue.

### 3.7 Flow cytometry confirms that metformin neutralizes radiation-induced immune cell infiltration

**Figure 6:**
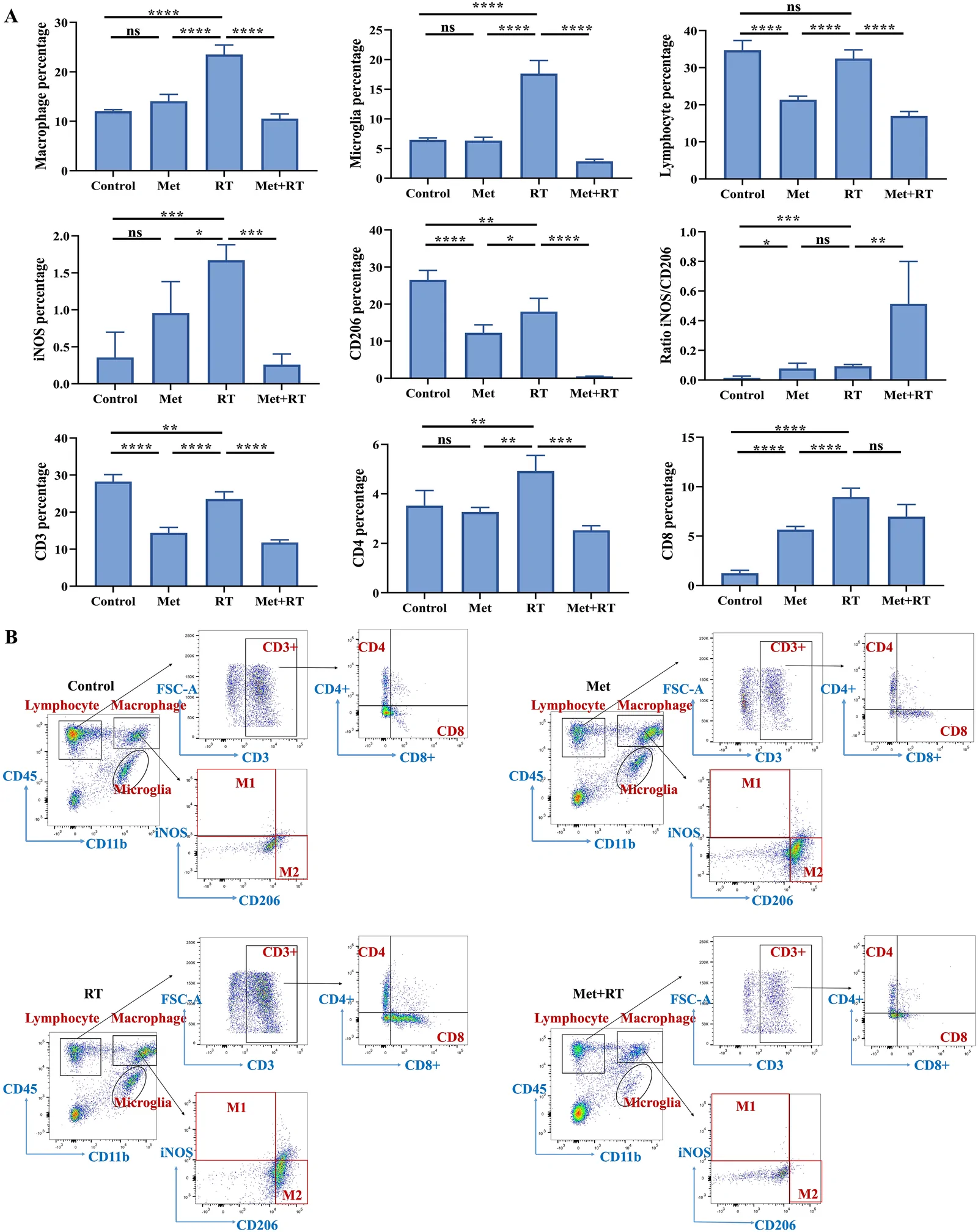
Metformin neutralizes RT-induced immune cell infiltration. C57BL/6 mice bearing intracranial CT-2A tumors were treated with saline, metformin, RT, or Met+RT (n = 15 mice per group at study initiation). On day 21, brain tumors from a subset of mice (n = 2 per group) were harvested, and mononuclear cells were isolated by density gradient centrifugation for multicolor flow cytometry analysis. **(A)** Flow cytometric quantification. Percentages of macrophage, microglia, Lymphocytes, CD3^+^ cells, CD3^+^ gated CD4^+^ and CD8^+^ subsets, iNOS^+^(M1), and CD206^+^(M2) are calculated in all live cells. **(B)** Gating strategy for the identification of lymphocytes, macrophages, and microglia cells isolated from mouse brain tumors. Representative flow plots schema for each group is displayed. Immune cells were also stained for CD45 and CD11b. Lymphocytes were defined as CD45^high^ CD11b^negative^ (CD45^hi^ CD11b^-^) cells. Infiltrating macrophages as CD45^high^ CD11b^positive^ (CD45^hi^ CD11b^+^) cells, and resident microglia as CD45^intermediate^ CD11b^positive^ (CD45^int^ CD11b^+^) cells. Gated CD3-positive events were analyzed for CD4 and CD8 production. M1 and M2 macrophages are characterized by iNOS and CD206 expression in CD45^hi^ CD11b^+^ macrophages. The data shown are from one experiment that is representative of two experiments. Data are represented as mean ± SEM; n = 2; *p <0.05, **p < 0.01, ***p < 0.001, ****p < 0.0001; n.s., not significant.

Flow cytometry was used to evaluate T cells and macrophages separately in live cells isolated and dissociated from the tumor (**Figure 6**) and non-tumor regions of the brain (**Supplementary Figure 4**). Consistent with the IHC results, a large percentage of TAMs (CD206^+^) relative to M1 macrophages (iNOS^+^) (> 70:1) and low infiltration of CD8^+^ T cells were observed in tumors from the control group (**Figure 6A**). Contrary to the presence of a large portion of macrophages in the tumor tissue, microglia were found in most of the non-tumor parts of the brain (**Supplementary Figure 4**). Metformin alone decreased the proportion of TAM (CD206) and increased CD8^+^ T cell infiltration without significantly changing M1 macrophage and CD4^+^ T cells. RT alone significantly increased total macrophages (CD45hi CD11b^+^) and M1 macrophages, increased the iNOS/CD206 ratio, and attracted more CD8^+^ T cells and CD4^+^ T cells compared with the control group in both the tumor and non-tumor regions of the brain (**Figure 6A**; **Supplementary Figure 4**). Dual treatment significantly decreased TAM by depleting CD206+ M2*Φ* and neutralized the M1*Φ*-inducing effect of RT, while overall markedly increasing the iNOS/CD206 ratio. Furthermore, Met+RT treatment promoted CD8+ T-cell infiltration while diminishing the RT-induced effects of immune cell infiltration to TME in total macrophages, total microglia, and total lymphocytes (**Figures 6A, B**). Overall, the therapeutic efficacy of RT for glioblastoma was enhanced by metformin which may have contributed to the anti-tumoral effects while simultaneously controlling the toxicities from the treatment by alleviating the RT-induced prominent inflammatory response.

### 3.8 Metformin promotes anti-tumor peripheral immune phenotype in combination with radiation

**Figure 7:**
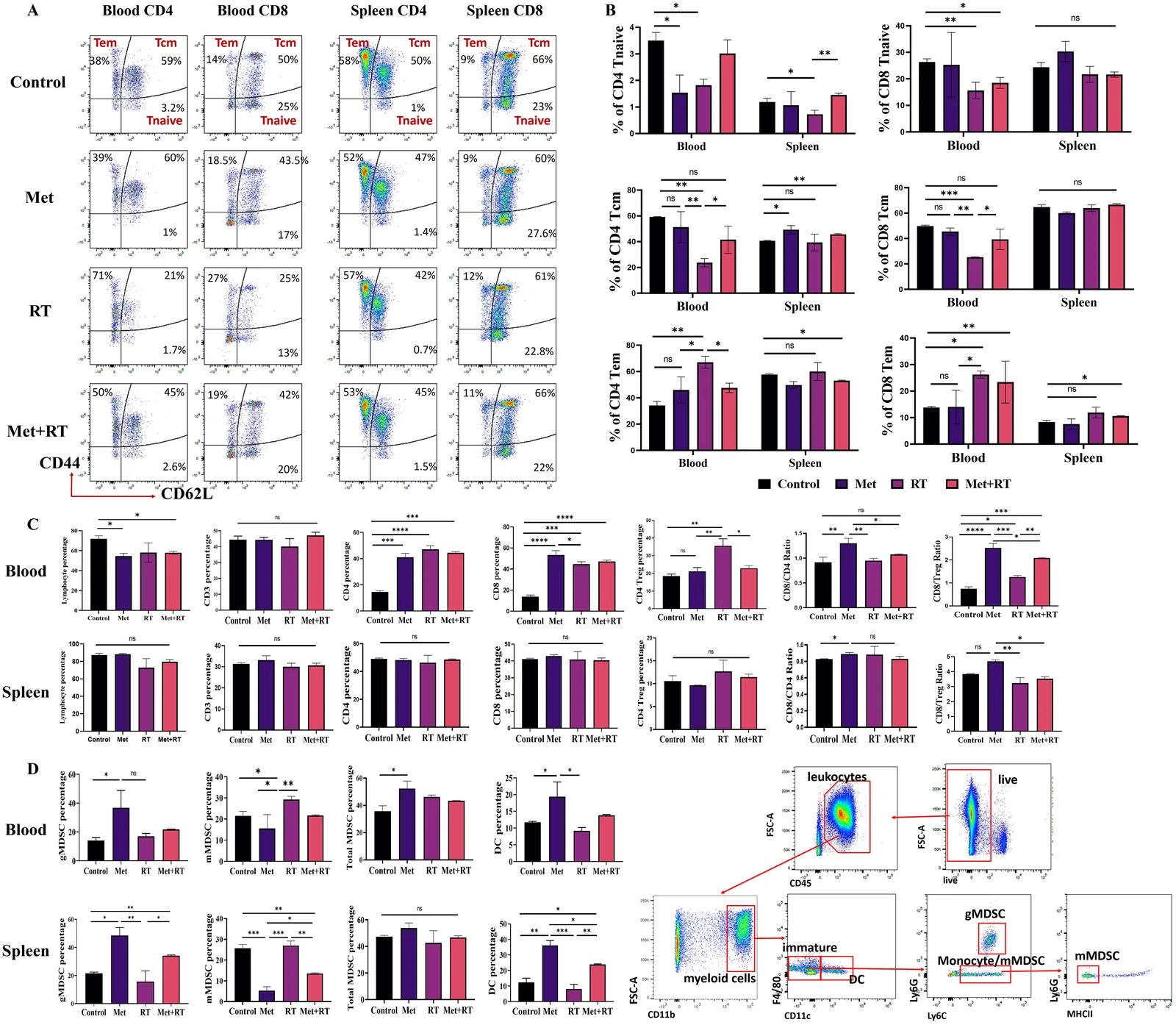
Metformin, when combined with radiotherapy, promotes an anti-tumor peripheral immune phenotype in GBM-bearing mice. C57BL/6 mice bearing orthotopic CT-2A tumors were treated with saline (Control), metformin (Met), radiotherapy (RT), or Met+RT. Peripheral blood and spleens were harvested, and immune populations were analyzed by multicolor flow cytometry. **(A)** Representative flow cytometric plots of CD4α and CD8α T-cell subsets, including naïve (Tnaive), central memory (Tcm), and effector memory (Tem) cells, defined by CD44 and CD62L expression status in blood and spleen.**(B)** Quantification of T-cell memory subsets showing that Met+RT was associated with an increased frequency of CD4α and CD8α effector memory T cells, particularly in peripheral blood, with more modest effects observed in the spleen. **(C)** Frequencies of lymphocytes, CD3α, CD4α, CD8α, and regulatory T cells (Treg), along with CD8/CD4 and CD8/Treg ratios in blood and spleen. While total CD3α T cells remained unchanged, metformin alone or combined with RT increased circulating CD4α and CD8α T cells and resulted in higher CD8/CD4 and CD8/Treg ratios, most prominently in blood. **(D)** Analysis of myeloid populations demonstrates RT-induced accumulation of monocytic MDSC (mMDSC) in blood and spleen, which was reduced by metformin alone and attenuated in the Met+RT group. Complete opposite responses to metformin and RT were observed in granulocytic MDSC (gMDSC). Representative gating strategies for leukocytes, myeloid cells, dendritic cells, and MDSC subsets are shown. Data are presented as mean ± SEM. *P <0.05, **p < 0.01, ***P < 0.001, ****p < 0.0001; n.s., not significant.

To study the peripheral immune response to metformin and RT, mice were euthanized, and peripheral blood and peripheral lymphoid organ, spleen, were harvested, and the immune profile was examined by flow cytometry. Metformin and RT, alone or in combination, did not alter the total number of CD3^+^ lymphocytes in the blood and spleen. All three treatments significantly increased the proportions of CD4^+^ and CD8^+^ T cells in peripheral blood. RT significantly increased the accumulation of T regulatory cells in the blood, a change that was offset by metformin treatment. Both metformin alone and Met+RT could substantially increase the CD8/CD4 ratio and the CD8/T regulatory cell ratio in peripheral blood. By contrast, CD4, CD8, and Treg cells in the spleen did not respond to metformin as much as in the blood, whereas the CD8/CD4 and CD8/Treg ratios in the spleen remained elevated by metformin treatment (**Figure 7C**).

The short-term memory in the peripheral immune system of mice that responded to the treatment was further studied by categorizing CD4 and CD8 T cells into central memory (Tcm), effector memory (Tem), and naïve memory (Tnaive) phenotypes based on CD62L and CD44 expression. The combined treatment did not affect naïve CD4 T cells in the blood or spleen but moderately decreased naïve CD8 naïve cells in the blood compared with control. For central memory cells, metformin combined with RT didn’t affect blood CD4 Tcm or blood and spleen CD8 Tcm, but it promoted CD4 Tcm in the spleen more than control, similar to the effect of metformin alone. Notably, when combined with RT, metformin significantly enhanced the expansion of CD4+ and CD8+ Tem cells in the blood compared with control, but the effects seem to be more moderate than the effects of RT alone (**Figures 7A, B**).

Circulating myeloid-derived suppressor cells (MDSC), particularly monocytic MDSC (mMDSC), have been reported to be positively associated with cancer stage and tumor burden across multiple cancers [24]. RT may induce MDSC accumulation, which in turn predicts RT failure and immunotherapy failure [25, 26]. Our results also showed that RT increased mMDSC accumulation in blood and spleen. By contrast, metformin alone reduced mMDSC levels in the blood and spleen of mice, and when combined with RT, it attenuated RT’s stimulatory effect on mMDSC in the peripheral environment (**Figure 7D**).

Additionally in **Table 2**, we summarize the effects of metformin on tumor cells, macrophage markers, cytokines/chemokines, tumor-infiltrating immune cells, peripheral T-cell subsets, and MDSC populations across all *in vitro* and *in vivo* experimental systems to clarify the context-dependent effects observed across the study (**Table 2**).

**Table 2:** Summary of metformin-associated effects across *in vitro* and *in vivo* experimental systems.

Experimental context	Cell/compartment	Effective metformin condition	Main readouts	Direction of effect	Interpretation
Cell viability assay	BMDM	2mM to 50 mM, 1 to 7 days	Cell viability, IC50	Low toxicity at ≤2 mM short-term; cumulative toxicity at higher mM doses	Lower doses used for immune modulation were below strongly cytotoxic ranges
Cell viability assay	CT-2A, MGPP3 glioma cells	2mM to 50 mM, 1 to 5 days	Cell viability, IC50	Direct cytotoxicity required higher mM exposure	In vivo effects are unlikely to be explained only by direct tumor-cell killing
Unstimulated BMDM	BMDM	0.1 to 2 mM	iNOS, CD206	↑ iNOS-like features, ↓ CD206-like features, strongest at 1 to 2 mM	Metformin can shift basal macrophage phenotype toward a less M2-like profile
IL-4/IL-13 stimulation	BMDM	0.1 to 2 mM	iNOS, CD206	Reversed IL-4/IL-13-induced M2-like skewing	Metformin reduces immunosuppressive/M2-like polarization under M2-skewing stimuli
IL-4/IL-13 stimulation	BMDM culture medium	0.1 to 2 mM	CCL2, CCL5, CXCL10, CXCL1, TNF-α	Reversed or increased selected cytokine/chemokine levels	Supports partial restoration of inflammatory/immune signaling under M2-skewing conditions
LPS stimulation	BMDM	0.1 to 2 mM	iNOS, CD206	↓ LPS-induced pro-inflammatory activation, partial restoration toward baseline	Metformin dampens excessive inflammatory activation rather than further promoting M1-like polarization
LPS stimulation	BMDM culture medium	0.1 to 2 mM	CCL2, CCL5, CXCL10, CXCL1, TNF-α	Attenuated LPS-induced cytokine/chemokine secretion	Supports inflammation-limiting effect under strong inflammatory stimulation
High M-CSF M2-skewed model	BMDM	0.1 and 2 mM, 12 and 24 h	M1-related genes: CD86, Tlr2, Ptgs2, Socs3, IL-12b, Nos2	↑ selected M1-associated transcripts, especially at 2 mM and 12 h	Transcriptional shift toward M1-like features
High M-CSF M2-skewed model	BMDM	0.1 and 2 mM, 12 and 24 h	M2-related genes: IL-6, CD301, CD206, Dectin1, IL-10	↓ multiple M2-associated transcripts, with partial recovery for some genes at 24 h	Suppression of M2-like program is dynamic and time-dependent
High M-CSF M2-skewed model	BMDM culture medium	0.1 to 2 mM, 24 h	TNF-α, CCL5, CCL2, IFN-γ, IL-12, IL-6, GM-CSF	No clear increase in functional M1 cytokines, but M2-associated cytokines such as CXCL1, IL-6 and GM-CSF were significantly downregulated	Metformin suppresses M2-like cytokines but does not fully mature macrophages into functional M1 state
RNA-seq/GSEA	BMDM	0.1 and 2 mM, 12 and 24 h	Hallmark pathways; KEGG/Reactome signatures; homeostatic marker heatmap	Enrichment of regulatory/homeostatic programs	Supports “homeostasis” as balanced activation rather than one-way polarization
MGPP3 conditioned medium	BMDM	0.1 to 2 mM	iNOS, CD206	↑ iNOS and ↓ CD206 mainly at higher concentrations	Metformin counteracts HGG-like tumor-conditioned M2 skewing, but less strongly
PPC2 conditioned medium	BMDM	0.1 to 2 mM	iNOS, CD206	↑ iNOS and ↓ CD206 at lower concentrations	Metformin more effectively reverses LGG-like tumor-conditioned M2 skewing
MGPP3 or PPC2 transwell co-culture	BMDM	0.5 to 2 mM	iNOS, CD206	↑ M1-like and ↓ M2-like features, stronger with PPC2	Tumor context determines the magnitude of metformin response
RT-conditioned MGPP3 co-culture	BMDM	Metformin + 2, 8, or 20 Gy RT	iNOS, CD206	Enhances low-dose RT inflammatory shift, but dampens higher-dose RT-induced M1-like activation	Metformin acts as a bidirectional modulator depending on inflammatory intensity
CT-2A orthotopic model	Tumor burden and survival	Met, RT, Met+RT	IVIS, survival	Met alone no survival benefit; Met+RT improved survival and reduced tumor burden versus RT alone	Therapeutic benefit is strongest in combination with RT
GL261 orthotopic model	Tumor burden and survival	Met, RT, Met+RT	IVIS, survival	Met+RT prolonged survival and reduced tumor burden	Confirms combination benefit in a second syngeneic GBM model
Tumor IHC	Intracranial CT-2A tumors	Met, RT, Met+RT	iNOS, CD206, CD3, CD4, CD8, FoxP3, iNOS/CD206, CD8/FoxP3	Met increased iNOS/CD206 and CD8/FoxP3 ratios; Met+RT reduced RT-associated FoxP3/Treg signal and maintained an antitumor CD8/FoxP3 profile while moderating RT-associated inflammatory activation	Supports reduced immunosuppressive tumor immune profile
Tumor flow cytometry	Intracranial CT-2A tumors	Met, RT, Met+RT	Macrophages, microglia, lymphocytes, CD3, CD4, CD8, iNOS, CD206	Met decreased CD206+ TAMs and increased CD8+ T-cell infiltration; Met+RT decreased CD206+ TAMs, promoted CD8+ T-cell infiltration, and moderated RT-associated macrophage/microglial/lymphocyte infiltration	Supports tumor-local immune remodeling with context-dependent dampening of RT-associated inflammation
Peripheral blood and spleen	T cells	Met, RT, Met+RT	CD3, CD4, CD8, Treg, CD8/CD4, CD8/Treg	Total CD3 unchanged; circulating CD4 and CD8 increased; RT-induced Treg increase attenuated by Met; CD8/Treg ratio increased	Supports systemic shift toward antitumor immune balance
Peripheral blood and spleen	T-cell memory	Met, RT, Met+RT	Tnaive, Tcm, Tem	Met+RT increased CD4+ and CD8+ effector memory T cells, especially in blood	Suggest enhanced peripheral immune memory features
Peripheral blood and spleen	MDSC subsets	Met, RT, Met+RT	mMDSC, gMDSC	RT increased mMDSC while Met reduced/attenuated it; conversely, RT decreased or Met increased gMDSC	Supports reduction of monocytic immunosuppressive myeloid compartment

BMDM, bone marrow-derived macrophage; GBM, glioblastoma; GSEA, gene set enrichment analysis; HGG, high-grade glioma; IHC, immunohistochemistry; IVIS, *in vivo* imaging system; LGG, low-grade glioma; Met, metformin; mMDSC, monocytic myeloid-derived suppressor cell; gMDSC, granulocytic myeloid-derived suppressor cell; RT, radiotherapy; TAM, tumor-associated macrophage; Tcm, central memory T cell; Tem, effector memory T cell; T naïve, naïve T cell.

αTaken together, our results showed that metformin combined with RT reprogrammed the hostile peripheral immune system toward an anti-tumor environment by inducing the accumulation of cytotoxic CD8 T cells, as well as the CD8/CD4 and CD8/Treg ratios and promoting the expansion of effector memory cells. In addition, metformin effectively blocked the RT-induced mMDSC induction and reduced mMDSC levels in the blood and spleen of GBM tumor-bearing mice.

## 4 Discussion

The dynamic interaction of GBM cells with their tumor microenvironment (TME) is the major contributor to treatment resistance, metastasis, cancer progression, and relapse (16). Stromal and non-cellular components of the extracellular matrix functionally cooperate to modulate multiple hallmark cancer properties, including proliferative capacity, clonal heterogeneity, migratory behavior, and immunosurveillance, thereby promoting malignant growth and invasive capability (41). Glioblastoma-associated macrophages and microglia (GAMs) represent the predominant stromal cell population within the tumor mass and play a critical role in shaping the immunosuppressive microenvironment. Restoring immunological homeostasis through GAM reprogramming and reinstatement of lymphocyte cytotoxic function has emerged as a central therapeutic objective in GBM treatment. Convergence of preclinical and clinical evidence has demonstrated the diverse anti-tumor functions of metformin, largely attributable to context-dependent immunomodulatory effects (24). Our experimental data indicate that high-concentration metformin (2 mM) induces only modest reductions in glioma cell viability, resulting in approximately 20% cell death after 3 days of exposure. This is consistent with previous reported concentrations of metformin to preferentially suppress cancer stem cells (42). The concentration of metformin required to achieve 50% cell death is higher in glioma cells than in BMDM. Given that therapeutic plasma metformin concentrations in humans range from 3 to 15 μM, these findings suggest that metformin may function primarily as an anti-tumor agent *in vivo* through indirect immunomodulatory mechanisms that restore immune homeostasis rather than direct cytotoxic effects on malignant cells (28).

GBM is composed of multiple ontogenetically distinct myeloid populations in TME, including infiltrating BMDM and tissue-resident microglia. A growing body of evidence indicates that the polarization of myeloid cells in GBM constantly and dynamically changes in response to a different context, including the stage of tumor development, the physical location inside and around the tumor, the variety of TME in different subtypes of tumors, and different phases in the recruitment from peripheral to tumor TME (43). These dynamic transitions reflect macrophage plasticity and context-dependent responses to environmental signals rather than fixed, unidirectional states. Conventional *in vitro* approaches for assessing metformin-mediated macrophage repolarization typically involve inducing unidirectional polarization followed by metformin exposure to evaluate phenotypic changes (44, 45). In addition to macrophages’ high plasticity, the effects of metformin on the immune system may vary in complex ways depending on the pathological context (28). The effects of metformin on macrophage polarization and repolarization remain controversial across studies, reflecting variations in methodology and experimental design. Literature examining metformin’s context-dependent effects on macrophage phenotypes illustrates this complexity. Wei et al. demonstrated that both low and high concentrations of metformin effectively repolarized M2-like macrophages toward an M1 phenotype (46). Similar findings were made in the breast cancer context. Results showed that metformin treatment could increase macrophage expression of M1-related cytokines, IL-12 and TNF-α, and attenuate M2-related cytokines, IL-8, IL-10, and TGF-β, by activating AMPK-NF-κB signaling in cancer cells (47, 48). Conversely, Ding and colleagues reported that metformin reversed interleukin-13-induced M2 differentiation without affecting M1 polarization (49). However, other studies also revealed the immunosuppressive effects of metformin by promoting M2*Φ* and inhibiting M1*Φ* particularly in inflammatory disease conditions (28, 45). Collectively, these studies and additional investigations suggested that metformin functions as an immunomodulator balancing between M1*Φ* and M2*Φ* properties across multiple disease conditions, with the magnitude and kinetics of these effects varying substantially based on stimulus intensity, metformin concentration, timing of exposure, and the specific inflammatory milieu. Thus, rather than a uniform “M1-promoting” or “M2-inhibiting” agent, metformin may function as a context-responsive immunomodulator that engages stimulus-dependent mechanisms to rebalance macrophage phenotypic states toward configurations that restore immune homeostasis.

Indeed, our findings support a context-dependent immunomodulatory role for metformin across multiple *in vitro* and *in vivo* models that recapitulate both immunosuppressive and inflammatory microenvironments (**Table 2**). Rather than acting as a uniform promoter of M1 polarization or inhibitor of M2 polarization, metformin appears to modulate macrophage activation according to the prevailing environmental stimulus. In this study, metformin suppressed M2-associated phenotypes induced by IL-4/IL-13, high-dose M-CSF, and glioma-conditioned media, while attenuating excessive inflammatory activation triggered by LPS or high-dose RT-associated stimulation. These bidirectional effects suggest that metformin primarily functions to constrain extreme macrophage polarization rather than to impose a fixed activation state.

To describe this phenomenon, we use the term “macrophage homeostasis” in an operational sense, referring to the maintenance or restoration of a balanced activation state in response to diverse environmental cues. This property of metformin may be particularly relevant in treating glioblastoma, where the tumor microenvironment is characterized by profound myeloid-driven immunosuppression. To model this condition, we generated an M2-dominant macrophage state using high concentrations of M-CSF (46) and examined the effects of metformin under conditions designed to mimic the GBM microenvironment. Transcriptomic analyses demonstrated that metformin altered both M1- and M2-associated gene-expression programs at low and high concentrations, with effects detectable within 12–24 hours. Notably, complementary cytokine profiling revealed only partial induction of M1-associated functional programs, further supporting the notion that metformin does not simply convert M2 macrophages into M1 macrophages. Rather, it appears to attenuate maladaptive polarization and promote a more balanced activation state.

The magnitude of these effects varied according to microenvironmental context and was most pronounced when macrophages were cocultured with less aggressive glioma cells. Together, these findings highlight the plasticity of macrophage responses to metformin and suggest that its immunological effects are determined by the baseline activation state of macrophages and the surrounding tumor milieu. This context-dependent regulation may help explain the heterogeneous findings reported across literature and provides a framework for understanding how metformin may remodel the glioblastoma immune microenvironment.

RT has been used for the treatment of cancer for more than a century and is now recognized as an essential part of standard care for GBMs. Although the direct effects of RT on tumor cells are well recognized, the consequences for tumor-associated immune cells remain debated. Emerging evidence indicates that radiation-induced inflammatory responses and immunosuppressive effects compromise therapeutic efficacy and potentially promote tumor progression and recurrence (50, 51). Consequently, strategies to enhance radiation efficacy while mitigating off-target immunological effects warrant investigation, particularly through combination with complementary immunomodulatory approaches. Macrophage reprogramming has emerged as a promising therapeutic strategy, driven by advances in understanding macrophage ontogeny, phenotypic heterogeneity, and functional specialization. However, important questions remain regarding the efficacy of all macrophage-targeting approaches. Extensive preclinical evidence supports the use of metformin as an anticancer agent through its immunomodulatory effects on the tumor microenvironment (52, 53). One effect of metformin in the tumor immune microenvironment is its ability to reprogram M2-like TAMs toward an M1-like phenotype in several cancers, likely through promoting M0 macrophage as an intermediate step. However, prospective clinical trials investigating metformin in cancer patients have yielded disappointingly modest results (54–56). Approaches to optimize metformin’s anti-tumor efficacy remain under active investigation. In this study, we hypothesized that combining metformin with radiation therapy would synergistically enhance therapeutic efficacy by leveraging radiation’s direct cytotoxicity against tumor cells while simultaneously engaging metformin’s context-dependent immunomodulatory properties to attenuate radiation-induced immunosuppressive sequelae and restore systemic immune homeostasis. Our results demonstrated significant improvement with metformin combined with RT in the GBM mouse model, including extended overall survival, inhibition of tumor progression, and improved antitumor immune environment. Metformin combined with RT increased macrophage recruitment and lymphocyte infiltration in the GBM tumor region in comparison with control and metformin alone, while modulating immune cell phenotypes and counteracting the pro-inflammatory and Treg-promoting effects of RT alone, thereby restoring immune homeostasis, but overall maintaining the immunostimulatory effects of RT.

T cell depletion, dysfunction, and exhaustion induced by tumor cells, in turn, promote tumor biological activities. In GBM, immunosenescence of the CD4+ T cell compartment has been associated with poor prognosis (53). Substantial reductions in thymic volume, accompanied by lymphopenia, have been documented in both GBM patients and syngeneic mouse models, indicating systemic T-cell depletion in glioblastoma condition (13). It has been reported that metformin treatment triggered a systemic decline in the total immune cell population in non-cancer mice, whereas it increased CD4+ T-cell and CD8^+^ levels in the head and neck squamous cell carcinoma (HNSCC) mouse model when combined with immune checkpoint inhibitors (57). Recent investigations further demonstrate that metformin promotes CD8^+^ T cell survival, restores CD8^+^PD-1^+^ T cell effector function, and enhances glycolytic metabolism and bioenergetic capacity of splenic CD8^+^ T cells (58, 59). Our results suggested a marked increase in CD4^+^ and CD8^+^ T cells in the mouse peripheral blood after metformin alone and after combined metformin and RT treatment, with a rise in both the CD8^+^/CD4^+^ T-cell ratio and the CD8^+^ T-cell/Treg-cell ratio, indicating restoration of T-cell homeostasis. However, no significant changes were found in the spleen. Additionally, metformin combined with RT, but not metformin or RT alone, markedly increased CD8 effector T memory cells and CD4 effector T memory cells in both the spleen and blood, demonstrating that restoration of systemic immune homeostasis requires coordinated engagement of both direct cytotoxic (RT) and context-dependent immunomodulatory (metformin) mechanisms.

Myeloid-derived suppressor cells (MDSCs) are primary components of the tumor microenvironment and have emerged as major regulators of immune responses in cancer and as critical therapeutic targets. MDSCs are found elevated in the peripheral circulation and tumors in GBM patients, which are associated with poor GBM outcomes (2, 60). MDSCs comprise two functionally and morphologically distinct subsets: monocytic MDSCs phenotypically resemble monocytes, whereas polymorphonuclear MDSCs exhibit neutrophil-like characteristics (60). M-MDSC rapidly differentiates into TAMs and inflammatory DCs, function as inhibitors of immune responses and promoters of tumor progression (61). A study on the efficacy of RT in prostate cancer showed that radiation (3Gy × 5) could increase the number of MDSCs in the spleen, lymph nodes, and peripheral blood (62). Similar concerns were also found in other studies, indicating RT could potentially facilitate the development and accumulation of MDSC, leading to the failure of RT in cancer (63, 64). The impact of metformin on the regulation of MDSCs in tumors remains unclear. A mouse study of head and neck squamous cell carcinoma demonstrated that a short-course of metformin, when combined with anti-programmed death-1 checkpoint blockade, reduced granulocytic MDSCs in both tumor and splenic tissues. In contrast, chronic pretreatment with metformin for 4 weeks prior to tumor inoculation reduced tumor growth but increased circulating granulocytic MDSCs (57). By examining the mMDSC and gMDSC in the blood and spleen of GBM tumor-bearing mice, we found that RT alone substantially elevated the frequency of mMDSC in peripheral blood compared to controls. Combining metformin with RT attenuated this radiation-induced mMDSC elevation, reducing mMDSC levels below RT-alone levels and restoring myeloid homeostasis. To our knowledge, this is the first study to report the effects of metformin alone or in combination with RT on dynamic alterations in peripheral lymphocyte populations, particularly memory T cells and MDSCs, and their integrated contribution to the restoration of systemic immune homeostasis in a mouse glioblastoma model.

We also noticed stronger anti-tumor/RT-sensitizing effects of metformin with improved survival in those mice carrying less aggressive tumor cells (GL261) than those implanted with CT-2A cells known to generate more immunosuppressive TME. This may raise the challenges of using metformin in clinic treating HGG/GBM as a radiosensitizer with extensively immunosuppressive TME. This limitation of metformin may be overcome by further enriching the metformin concentrations in TME although that could be clinically challenging as well. Nevertheless, metformin may function well treating lower grade gliomas that are commonly treated with cytotoxic chemotherapy and RT (25–27).

Given the complexity of metformin’s context-dependent immunomodulatory mechanisms, we tested it in multiple *in vitro* conditions and two immunocompetent GBM models in combination with RT, a standard of care for GBM, and many other cancers. With the strength of the experimental design, we consistently observed the context-dependence of metformin effects in immune cells, particularly TAMs in GBM. However, the results of our study have limitations. Although we used two tumor models, both are highly relevant to the current GBM clinical setting and observed that the response to metformin treatment is disease dependent, the experimental findings do not fully encompass all clinical scenarios encountered in human disease with both animal models still known to not as representative as the profound immunosuppressive TME in patients’ tumor. Coculture experiment *in vitro* did not include other cell types in TME such as T cells and vasculature. The molecular mechanism of metformin’s context-dependent functions on TAM remains unclear. Metformin exhibits context-dependent systemic immune effects that are substantially modulated by the composition of the tumor microenvironment, timing and type(s) of concomitant therapeutic interventions, and individual metabolic and immunological status in the tumor. Our findings support the potential of metformin as a therapeutic agent to counteract immune cell dysfunction in response to RT. Additionally, metformin may reverse the immunosuppressive microenvironment of the tumor but counteracts extreme inflammatory conditions induced by RT or pyrogens, thereby facilitating a homeostatic immune response against the tumor.

## 5 Conclusions

To recapitulate, in the present study, we observed a diverse range of effects of metformin on macrophage polarization *in vitro*. Metformin’s effects on immune cells are context dependent and is most consistently as a homeostasis promoter rather than a one-directional promoting drug in macrophages. Thus, in extensively immunosuppressive GBM TME, it functions by suppressing M2-like but promoting M1-like tumor-associated macrophages but the effects are limited when used alone. In addition, the present results demonstrated a rational and effective combination of metformin and RT that can lead to sustained tumor control. Metformin attenuated the immunosuppressive effect of RT. Working together, metformin and RT treatment could increase immune cell infiltration and activate the cytotoxic T cells in the tumor area. Meanwhile, the combination can enhance the effects of T memory cells and CD8^+^ cytotoxic cells, while improving MDSCs in the peripheral system in the mouse GBM model.

## Data Availability

All data supporting this study are available from the corresponding author upon reasonable request. For the RNA-seq data used in the present study, the official GEO accession ID is GSE342855.

## Supplementary material

Supplementary Figure 1 GSEA profiling of mouse BMDMs following metformin treatment. Composite heatmaps illustrate the top regulated biological pathways in mouse bone marrow-derived macrophages (BMDMs) that were pre-induced to M2-like macrophages by m-CSF. The analysis highlights the most significantly regulated (top 5 up-regulated and top 5 down-regulated) pathways within the Hallmark, KEGG, and Reactome categories when comparing (A) control (Ctrl, no drug) versus Metformin (MET) treatment (0.1mM or 2mM) at 12h; (B) control versus MET treatment (0.1mM or 2mM) at 24h; and (C) 2mM versus 0.1mM Met treatment at 12h and 24h. The Normalized Enrichment Score (NES) represents the degree of over-representation of a pathway at the top (red) or bottom (blue) of the ranked gene list. Right Columns show the -log_10_ P-value. Pathways are grouped by their respective MSigDB categories and sorted by NES. (D) Advanced heatmap demonstrates increase in mRNA expression of representative genes in Homeostasis pathway with most prominent effects seen in cells treated with 2mM metformin for 12 hours (12h_2) with persistent effects seen in some genes particularly those in metabolism in cells treated for 24 hours with 0.1mM (24H_0.1) or 2mM (24H_2) drug, confirming the promoting effects of metformin on the homeostasis pathways.

Supplementary Figure 2 The combination of metformin and RT suppresses tumor growth in the orthotopic GL261 mouse model. (A) Time course of tumor implantation, metformin, and RT administration. GL261-luc glioma cells were implanted stereotactically into the right caudate putamen of 12-week-old female C57BL/6 mice. Eight days after inoculation, mice were treated daily with metformin (500 mg/kg in 100 μL PBS) by oral gavage for 5 weeks or until endpoint. Mice in selected group received RT (8 Gy) as monotherapy and combination therapy on day 11. (B) Kaplan-Meier survival curve of mice bearing GL261 tumor following treatment with control, metformin, RT, and the combination of MET and RT. Data were analyzed using the log-rank (Mantel-Cox) test; *p < 0.05, ***p<0.001, ****p < 0.0001, n.s., not significant. (C) Tumor growth of GL261 cells was indicated by mean fluorescence intensity in C57BL/6J mice treated with metformin, RT, and the combination. Data represent mean ± SD and n = 13 mice per group.

Supplementary Figure 3 IHC quantification of the non-tumor hemisphere of the mouse brains. Bar graphs represent the total number of positive cells per mm^2^ non-tumor tissue for iNOS, CD206, CD3, CD4, CD8, FoxP3, M1/M2 ratio, and CD8^+^/FoxP3^+^ ratio in control, Met, RT, and Met+RT groups; Bars represent mean ± SEM. Each symbol corresponds to one mouse, n=5; *P <0.05, **p < 0.01, ***P < 0.001, ****p < 0.0001; n.s., not significant.

Supplementary Figure 4 Flow cytometry quantification of cells from the non-tumor hemisphere of the mouse brains. C57BL/6 mice bearing CT-2A tumors were treated with saline (Control), metformin (Met), radiotherapy (RT), or Met+RT (n = 15 per group). On day 21, the non-tumor part of the mouse brains of 2 mice from each group were also harvested for flow cytometry analysis. Percentages of macrophage, microglia, Lymphocytes, CD3^+^ cells, CD3^+^ sorted CD4^+^ and CD8^+^ subsets, iNOS^+^(M1), and CD206^+^(M2) are calculated in all live cells. Data are represented as mean ± SEM; n = 2; *P <0.05, **p < 0.01, ***P < 0.001, ****p < 0.0001; n.s., not significant.
